# Supplementary material for: Stress-response balance drives the evolution of a network module and its host genome
Source: Mol Syst Biol. 2015 Aug 31;11(8):827. doi: 10.15252/msb.20156185 (PMC4562500; doi:10.15252/msb.20156185)

Appendix

Stress-response balance drives the evolution of a network module and its host genome

Caleb González[1*], J. Christian J. Ray[1,2,*], Michael Manhart[3,4], Rhys M. Adams[1], Dmitry Nevozhay[1,5], Alexandre V. Morozov[3,6], and Gábor Balázsi[1,7,8,‡]

[1]Department of Systems Biology - Unit 950, The University of Texas MD Anderson Cancer Center, Houston, TX 77054, USA

[2]Center for Computational Biology & Department of Molecular Biosciences, University of Kansas, Lawrence, KS 66045, USA

[3]Department of Physics & Astronomy, Rutgers University, Piscataway, NJ 08854, USA

[4]Department of Chemistry and Chemical Biology, Harvard University, Cambridge, MA 02138, USA

[5]School of Biomedicine, Far Eastern Federal University, 8 Sukhanova Street, Vladivostok, 690950, Russia

[6]BioMaPS Institute for Quantitative Biology, Rutgers University, Piscataway, NJ 08854, USA

[7]Laufer Center for Physical & Quantitative Biology, Stony Brook University, Stony Brook, NY 11794, USA

[8]Department of Biomedical Engineering, Stony Brook University, Stony Brook, NY 11794, USA

[*]Equal contribution

Table of Contents

1. Evolution experiments and sequencing: Experimental procedures 3
   1.1. 12-hour resuspension experiments 3
   1.2. 24-hour resuspension experiments 3
   1.3. Flow cytometry 3
   1.4. Whole-genome sequencing 3
   1.5. Sanger sequencing 4
   1.6. Primers used for sequencing extra-circuit mutations 5
   1.7. Primers used for sequencing the rtTA (regulator) region 5
   1.8. Primers used for sequencing the yEGFP::zeoR (reporter) region 6
   1.9. Primers used for reconstructing mutations in the ancestral genome 6
2. Sequencing: Data analysis 7
   2.1. Overview 7
   2.2. Structural variant reconstruction and realignment 8
3. Computational and mathematical models of PF circuit and evolutionary dynamics 9
   3.1. Model of PF cell dynamics and parameter estimation 9
   3.2. Mathematical model of evolutionary dynamics 12
   3.3. Evolutionary simulation framework 15
   3.4. Steps in the algorithm 17
   3.5. Justification of parameter choices 19
4. Extended results of evolutionary dynamics simulations 19
   4.1. The effects of various parameters on evolutionary dynamics 19
   4.2. Agreement between predicted and observed evolutionary dynamics 20
5. Appendix References 21
6. Appendix Figure Legends 22
7. Appendix Tables 25

1. Evolution experiments and sequencing: Experimental procedures

1.1. 12-hour resuspension experiments

We started by determining the cell concentration of overnight cultures. Then we diluted the cells to 10^6 cells/mL and inoculated them into six different treatments based on SD-*his-trp* medium with 2% galactose: D0Z0 consisting of neither Doxycycline nor Zeocin; D2Z0 consisting of Doxycycline only (2 µg/mL; Acros Organics, Geel, *Belgium*); DiZ0 consisting of 0.2 µg/mL Doxycycline only; D0Z2 consisting of only Zeocin (2 mg/mL; Invitrogen, Carlsbad, CA); D2Z2 consisting of 2 µg/mL Doxycycline and 2 mg/mL Zeocin; and DiZ2 consisting of 0.2 µg/mL Doxycycline and 2 mg/mL Zeocin. 10^6 cells were then resuspended similarly into their respective treatments every 12 hours for ~3 weeks. Cell density was measured (Nexcelom M10) every 12 hours and fluorescence was measured by flow cytometry every 24 hours. Malthusian fitness between subsequent resuspensions was estimated by a linear fit to log-transformed total cell counts (inferred from cell density and culture volume): $g = \frac{1}{12}\ln\left(\frac{N_{12}}{N_0}\right)$. Samples were saved daily and stored in 80% glycerol at -80°C for further studies.

1.2. 24-hour resuspension experiments

Overnight cultures were diluted 100-fold, divided into 3 replicates, each inoculated into four different treatments: D0Z0, D0Z2, D2Z0, and D2Z2, as described above. Cultures were re-suspended daily into their respective treatments with appropriate dilutions aiming to start each time at 10^6 cells/mL. Cell density was measured daily on a Nexcelom M10 cell counter (Nexcelom Bioscience). The logistic growth equation $\frac{dN}{dt} = gN\left(1 - \frac{N}{N_{\max}}\right)$ was then used to estimate the Malthusian fitness $g = \frac{1}{24}\ln\left(\frac{N_{24}}{N_0} - \frac{N_{24}}{N_{\max}}\right) - \frac{1}{24}\ln\left(1 - \frac{N_{24}}{N_{\max}}\right)$ between subsequent resuspensions. Samples were saved daily and stored in 80% glycerol at -80°C for further studies.

1.3. Flow cytometry

Cultures were analyzed on a FACSAria II flow cytometer and sorter (BD Biosciences, San Jose, CA) using the 488nm blue excitation laser and 530/30 emission filter for EGFP. Raw flow cytometry data files were read into MATLAB (Mathworks, Inc.) using the Matlab script fca_readfcs (MATLAB Central) for plotting and analysis. We divided the fluorescence intensity (FL1) by (FSC)¾ for an approximate normalization by cell volume to approximate yEGFP concentrations, minimizing cell-cycle and cell size biases. The unnormalized data was very similar, except the distributions were generally broader.

1.4. Whole-genome sequencing

Samples taken directly from the -80°C collection were inoculated into the same treatment in which the cells evolved. After overnight shaking at 30°C, whole-genome extractions were performed with the DNeasy Blood & Tissue Kit (Qiagen) using the supplier's protocol modified for yeast. Genomic

extracts were treated with 2 µg of RNAse (Qiagen) for 75 minutes at 37°C. Samples were then whole-genome sequenced at MD Anderson's Sequencing and Microarray Facility on a HiSeq2000 Sequencer (Illumina Inc.). The average coverage genome-wide was ~100. Allele frequencies were inferred as: $N_{(\text{reads with the allele})}/N_{(\text{total reads at that locus})}$.

1.5. Sanger sequencing

Samples were streaked on plates as described. Ten to twelve single well-isolated colonies were picked and incubated overnight shaking at 30°C in 1.5 mL SD-*his-trp* medium. The following day 0.5 mL of overnight culture was saved in 80% glycerol at -80°C for further studies. The remaining 1 mL of overnight culture was used for genomic extractions as described above. Sanger sequencing was performed on a Seqwright DNA sequencer (GE Healthcare / Seqwright Genomic Services, Houston, TX), utilizing specific primers that targeted the region of interest (primer list below). Sequences were aligned to the reference sequence and analyzed using the SEQMAN PRO software (DNASTAR®, Lasergene). Sequencing primers were designed to target the genetic sequence with at least double coverage, and mutation calls were confirmed with at least two different shotgun sequences resulting from the respective primers. Allele frequencies were inferred as: $N_{(\text{colonies with allele})}/N_{(\text{total colonies})}$.

1.6. Primers used for sequencing extra-circuit mutations

Name	Sequence
Gal2-1-Seq-f	GGTTTAATCGTCGCCATTTTCA
Gal2-1-Seq-r	GGAACTAACGTCAAAGCGCC
GAL2-Seq-f	ATGGCCGTAAAAAGGGTCTTTCGATTGTCG
GAL2-Seq-r	GGAGGCAAAGTTGACTACACCAATGACAATG
EOS1-Seq-f	GCAAGAGCGTCTGAATATCT
EOS1-Seq-r	CTGTCGTTGGAAGGAACATC
MLH3-f	GCGCTCGCAAATAAACGCAAACTAAATATAAGCG
MLH3-r	GTTAGTCTCTGCTGAACATCAATCTCAGATGATTC
STB3-Seq-f	TAATGTGTCTAATGGCCAATGGTCCCATTTC
STB3-Seq-r	GATTATAAAAGACACTGATTGCAAACGGTGC
YDR186c -Seq-f	TGTCATTTAATAATTTGGAAAATGCGTTTG
YDR186c -Seq-r	ACGATGAAGGTTTGAGCTTTTACGCTCCAAGC
INO2-Seq-f	CTGCTGAGCATTTGGTAAGCAGTTTCAAAG
INO2-Seq-r	AACCACACTCAAACCAAATCTACGCCAGCTAC
RPS0A-Seq-f	GGTTTAATCTGGTACTTATTGGCTAGAGAAG
RPS0A-Seq-r	TTTAGATCACTATATACGCTACGTGTTGATAAG
SRX1-Seq-f	AGCCTTTGGCCTCTGCAATACTAGCGATTGC
SRX1-Seq-r	GCCTGTTCTAGAGAGCATGTCTTGCTAGCGG
RPL9B-Seq-f	ATCGTCAAGGTTGTCGGTCCAAGAGGTACTTTG
RPL9B-Seq-r	GTCTTCGACAATGAAACCCTTGTGGGAAACG
GUP2-Seq-f	ACTTACGCGCCATTGTTTTTAGTGGGCCCAATTATCAC
GUP2-Seq-r	TTGGATGTCATGCCATATTGCTACAAATGAGAATAC

1.7. Primers used for sequencing the rtTA (regulator) region

rtTA amplifying primers:

Name	Sequence
TRP-f	ATGTCTGTTATTAATTTCACAGGTAGTTC
DED1-r	TCCATCTACCACCAGAACGGCCGTTAGATC

rtTA sequencing primers:

Name	Sequence
rtTA-seq-int-r-cg	CGACTTGATGCTCTTGTTCTTCCAATACGCAACC
rtTA-seq-int-f-cg	GCCAACAAGGTTTTTCACTAGAGAATGCATTATATG
Tetreg-AflII-f	GCGCCTTAAGGCGCCACTTCTAAATAAGCGAATTTC
rtTABamHI2-f	GCGCGGATCCATGTCTAGATTAGATAAAAGTAAAG
FFF-XhoI-r	GCGCCTCGAGTTAACCTGGCAACATATCTAAATCAAAGTCATC
	TAATGCGTCGGCGGGTAGCATGTCTAGGTCGAAATCGTCAAGAGACA
Backbone-r	CGCGTTGGCCGATTCATTAATGC

1.8. Primers used for sequencing the yEGFP::zeoR (reporter) region

yEGFP::ZeoR amplifying primers:

**Name**	**Sequence**
His-f	ATGACAGAGCAGAAAGCCCTAGTAAAGC
Before2TRP-r	CACATATATTACGATGCTGTTCTATTAAATGCTTCC

yEGFP::ZeoR sequencing primers:

**Name**	**Sequence**
Backbone-r	CGCGTTGGCCGATTCATTAATGC
GalSeqE-r	TGAATAATTCTTCACCTTTAG
ZeoR-XhoI-r	gcgcctcgagTCAGTCCTGCTCCTC
yEGFP-BamHI-f	gcgcggatcctattaaaATGTCTAAAGGTGAAG
Origin-Middle-f	CTATTAAAGAACGTGGACTCCAACGTCAAAG
Tetreg-AflII-f	GCGCCTTAAGGCGCCACTTCTAAATAAGCGAATTTC

1.9. Primers used for reconstructing mutations in the ancestral genome

Mutation #1 in D2Z2 ($rtTA_{+225}$)

**Name**	**Sequence**
rtTA-mod1-f	GCCCTTTAGAAGGGGAAAGCTGCCAAGATTTTTTACGTAATAACGC
rtTA-mod1-r	GCGTTATTACGTAAAAAATCTTGGCAGCTTTCCCCTTCTAAAGGGC

Mutation #2 in D2Z2 ($rtTA_{-9}$)

**Name**	**Sequence**
Tetreg-AflII-f	GCGCCTTAAGGCGCCACTTCTAAATAAGCGAATTTC
Cyc1-BamHI_mod-r	GCGCGGATCCCCGGAATTGATCCGGTAATTTAGTG

2. Sequencing: Data analysis

2.1. Overview

We performed whole-genome sequencing with Illumnia HiSeq as described in the Experimental Procedures, obtaining 76-bp paired-end reads. We applied the following analysis pipeline:

1: Align reads (BWA/Bowtie 2)
2: Remove PCR duplicates (SAMtools)
3: Recalibrate base quality (GATK)
4: Calculate base alignment quality (SAMtools)
5: Call variants in each sample (FreeBayes)
6: Filter S288c/YPH500 strain differences
7: Match variants across time points
8: Manually validate in alignment (IGV)

We first aligned the raw reads to the S288c genome (*Saccharomyces* Genome Database, build R64-1-1) with the synthetic PF sequence inserted into chromosome XV (S288c+PF). To reduce biases from the alignment algorithm, we used both BWA-MEM (Li, 2013) and Bowtie 2 (Langmead & Salzberg, 2012) on default settings; we confirmed all reported mutations in both alignments, although for simplicity we only show the results from the BWA alignment.

We filtered the aligned reads for PCR duplicates using SAMtools (Li et al, 2009). We recalibrated raw base qualities from the sequencing instrument in two ways. First we recalibrated them in GATK (DePristo et al, 2011; McKenna et al, 2010) using a sample-specific error model accounting for biases in nucleotide and dinucleotide identity and position on the read. We then used SAMtools (Li et al, 2009) to cap the resulting base qualities by their read mapping quality ("base alignment quality") to suppress mismatches resulting from misalignments near short indels

We called SNPs using a heuristic filtering scheme implemented in FreeBayes (Garrison & Marth, 2012). For extra-PF variant calling, we filtered reads with Phred-scaled mapping quality less than 1 (to eliminate reads mapping to multiple locations) and bases with quality less than 10. We required putative variants to be supported by at least 2 reads and at least 5% of all reads covering that base; total coverage at the site is required to be greater than 30 (average coverage genome-wide is ~100). For variant calling within the PF sequence, we did not filter reads based on mapping quality because important parts of the PF sequence are repeated (e.g., the promoters for yEGFP::zeoR and rtTA), and because we could more easily assess possible alignment problems by hand. In all cases we also eliminated variants supported by reads mapping only to a single strand.

The above steps produced a list of putative variants for each sample. Most of these are likely due to systematic differences between the S288c genome and the genome of the YPH500 strain used in our experiments. To eliminate these, we only considered extra-PF variants that appeared in a single experiment (i.e., the same variant appearing in different experiments was presumed to be spurious) and at multiple time points within the same experiment. We also rejected variants in regions with dubious alignment (e.g., telomeres, transposons, long repeats). We did not apply these criteria to variants in the PF sequence. We also required all variants to be detected in both the BWA and Bowtie 2 alignments.

Finally, we manually validated all variants using the Integrative Genomics Viewer (Robinson et al, 2011; Thorvaldsdottir et al, 2013). **Appendix Tables S2-S5** list all such mutations passing these conditions.

2.2. Structural variant reconstruction and realignment

Sanger sequencing detected five larger structural variants (one 30-bp duplication, three 42-bp deletions, and one 78-bp deletion) in the synthetic PF sequence. Since our analysis pipeline for the whole-genome sequence data is not equipped to detect these, we attempted to confirm them in the whole-genome data by reconstructing the variants in the wild-type reference genome and then realigning the reads.

For the 30-bp tandem duplication detected in 24hr-D2Z0-r1 (**Appendix Table S3**), the alignment to the wild-type reference shows a clear signature of the duplication in the coverage profile (**Appendix Figure S4D**, top). As expected, when we aligned these same reads to a reference with the 30 bp duplication included, the coverage enhancement disappeared (**Appendix Figure S4D**, bottom). However, the reads did not align perfectly to this modified reference sequence because the duplication is only present in a fraction of the population.

In contrast to the tandem duplication, there was no clear signature of the three 42 bp promoter deletions (in 12hr-DiZ0-r1, 24hr-D0Z2-r1, and 12hr-DiZ2-r1) in the alignments to the wild-type reference, based on coverage profiles and anomalous mate pairs. Still, the reads aligned without major problems to reference genomes with the deletions included (not shown), supporting the validity of the deletions. Finally, we considered the 78 bp deletion in the activator domains of rtTA (occurring in condition 24hr-D2Z0-r3, **Appendix Table S3**). The alignment to the wild-type reference near the 78 bp deletion produced a spurious point mutation (**Appendix Figure S4E**, top), which was not resolved by aligning to a reference with the deletion included (**Appendix Figure S4F**, bottom). Moreover, the new alignment produced a signature of enhanced coverage reminiscent of a duplication; this is because the 78 bp deletion eliminates two of the three activator domains, but since most of the population (~70%) does not have the deletion, their reads appear to have a duplication relative to a reference that includes the deletion.

3. Computational and mathematical models of PF circuit and evolutionary dynamics

In this section we introduce mathematical models capturing the dynamics of PF cells and the evolutionary dynamics of PF cell populations. In section 3.1, we describe a model of the dynamics of intact PF cells prior to experimental evolution. The subsequent two sections describe our two models of evolutionary dynamics. The first of these is an ordinary differential equation (ODE) model that describes how the number of cells with wild-type and various mutant genotypes vary over time, based on their fitness. The second is a more detailed model that explicitly accounts for phenotypic switching and resuspensions during our evolution experiment.

3.1. Model of PF cell dynamics and parameter estimation

We consider a model of PF cell dynamics described in previous work (Nevozhay et al, 2012). We defined the cellular fitness function $g(Z,D,F)$ that describes how the division rate of cells with gene expression (fluorescence) *F* depends on extracellular Zeocin (*Z*) and Doxycycline (*D*) concentrations. In the function $g(Z,D,F)$, *F* represents both rtTA and yEGFP::zeoR protein levels, which we assumed to be proportional (since they are expressed from identical promoters). Cellular fitness $g(Z,D,F)$ was assumed to be a product of a constant (g_0) and two functions: the Zeocin effect $\gamma_Z(Z,F)$ and the Doxycycline effect $\gamma_D(D,F)$, each obtained from simple biochemical considerations. The population fitness is obtained by averaging the cellular fitness over the gene expression distribution $p(F)$, $G(Z,D) = \langle g(Z,D,F) \rangle_F = \int_F g(Z,D,F)p(F)dF$.

First, we considered the Zeocin effect $\gamma_Z(Z,F)$ to be proportional to the amount of DNA undamaged by Zeocin binding. Damaged DNA arises from intracellular Zeocin binding to DNA, and is constantly repaired at a fast rate. Undamaged DNA thus depends inversely on the amount of intracellular Zeocin, $Z_i(Z,F)$. We model the Zeocin effect in the following way:

$$\gamma_Z(Z,F) = \frac{\chi}{Z_i(Z,F) + \chi}. \quad (1)$$

where χ is the Zeocin-DNA association constant. Note that the intracellular Zeocin concentration Z_i is always smaller than the extracellular Zeocin concentration Z. The rate of change for intracellular Zeocin concentration Z_i depends on the balance between Zeocin gain and loss. Zeocin gain equals the influx from extracellular medium ϕZ, where ϕ is the rate of extracellular Zeocin influx. Zeocin loss equals the outflux/dilution $h_z Z_i$ (h_z is the combined outflux/dilution rate of internal Zeocin) and the flux of yEGFP::zeoR binding to Zeocin, sRZ_i (s is the rate of Zeocin binding to yEGFP::zeoR), which converts the free form of this protein, *R*, into the Zeocin-bound form, *B*. We obtain the following rate equation for intracellular Zeocin:

$$\dot{Z}_i = \phi Z - h_z Z_i - sRZ_i. \quad (2a)$$

Likewise, the rate of concentration change for the Zeocin-bound form, *B*, of yEGFP::zeoR is:

$$\dot{B} = sRZ_i - gB, \quad (2b)$$

where g is the cellular growth rate that causes protein dilution.

Together with the constraint $R+B=F$ (total yEFGP::zeoR, consisting of free and unbound forms), at steady state we obtain from (2a) and (2b) the quadratic equation:

$$sh_z\hat{Z}_i^2 + (gh_z + sgF - s\phi Z)\hat{Z}_i - g\phi Z = 0 \quad (2c)$$

We solve (2c) to find the steady state of $Z_i(Z,F)$, $\hat{Z}_i$. This quasi-steady state approximation of these processes gives the Zeocin effect $\gamma_Z(Z,F)$ in the form:

$$\gamma_Z(Z,F) = \frac{\chi}{\hat{Z}_i(Z,F)+\chi}. \quad (3)$$

We estimated the value of the Zeocin-DNA association constant χ considering that the growth rate in Zeocin (D0Z2) is approximately $g_0/2$. Since $\phi Z/h_z$ is the steady-state intracellular Zeocin concentration (**Eqn. 2a**), we obtain $\chi = \phi Z/h_z$.

We next consider the effect of Doxycycline on fitness, $\gamma_D(D,F)$. We assumed that active Doxycycline-bound rtTA (denoted A) has a first-order Hill-type inhibitory contribution to the Doxycycline effect $\gamma_D(D,F)$. Similar to the Zeocin effect, we calculated $\gamma_D(D,F)$ considering that this toxic form of rtTA arises from Doxycycline binding to free rtTA:

$$\gamma_D(D,F) = \frac{\alpha}{\alpha + A} = \frac{\alpha}{\alpha + F\dfrac{D}{D+\beta}}, \quad (4)$$

where the parameter α describes the toxicity of activated rtTA, and β describes the binding efficiency of rtTA to Doxycycline.

To estimate the parameters s, ϕ, g_0, α, and β (note that h_z is not a free parameter as we estimated it *a priori*), we performed nonlinear fitting by minimizing the objective function

$$\Omega = \sum_{D,Z}\Big(G_E(D,Z) - g_0\big\langle\gamma_Z(Z,F)\gamma_D(D,F)\big\rangle\Big)^2, \quad (5)$$

where the sum is over the 13 Doxycycline and Zeocin concentrations (indicated by crosses below) at which we measured population fitness G_E (**Appendix Figure S1A**):

	Dox=0.0 μg/mL	Dox=0.2 μg/mL	Dox=0.5 μg/mL	Dox=1.0 μg/mL	Dox=2.0 μg/mL
Zeo=0.0 mg/mL	✖	✖	✖	✖	✖
Zeo=0.5 mg/mL	✖				
Zeo=1.0 mg/mL	✖				
Zeo=1.5 mg/mL	✖				
Zeo=2.0 mg/mL	✖	✖	✖	✖	✖

The resulting parameter values are given in **Appendix Table S1**.

Once known, we can use the cellular fitness function $g(Z,D,F)$ to predict the cellular and/or population fitness levels at arbitrary concentrations of Doxycycline and Zeocin. Since the distribution of fluorescence levels (expression) tends to be tightly concentrated around a high value, a low value, or both (bimodal), we can use the inferred cellular fitness function and the fluorescence distribution to coarse-grain the model into two effective expression states. We define high (H) and low (L) expressor populations relative to an expression threshold *θ*. Then the average fitness of high and low expressor cells is

$$g_H(Z,D) = \langle g(Z,D,F) \rangle_{F>\theta} = \int_{F>\theta} g(Z,D,F)p(F)dF, \qquad (6a)$$

$$g_L(Z,D) = \langle g(Z,D,F) \rangle_{F<\theta} = \int_{F<\theta} g(Z,D,F)p(F)dF. \qquad (6b)$$

Knowing the fitness values g_H and g_L, the switching and growth of H and L cells are described by the system of equations:

$$\begin{cases} \dfrac{dL}{dt} = g_L L + fH - rL \\ \dfrac{dH}{dt} = g_H H - fH + rL \end{cases} \qquad (7a)$$

where *r* is the "rise" rate of switching from low to high expression, and *f* is the "fall" rate of switching from high to low expression. From these equations we can calculate the values of the population growth rate g_T, and the ratio of *H* and *L* cell fractions R_∞ after the initial transient period has passed:

$$g_T = \frac{g_L - r + g_H - f + \sqrt{(g_H - f - g_L + r)^2 + 4rf}}{2} \tag{7b}$$

$$R_\infty = \frac{L}{H} = \frac{2f}{h - l + \gamma} = \frac{l - h + \gamma}{2r} = \frac{2f}{g_H - f - g_L + r + \sqrt{(g_H - f - g_L + r)^2 + 4rf}} \tag{7c}$$

Since we can determine R_∞ after directly measuring the number of H and L cells (by flow cytometry and cell counting), as well as the overall population growth rate g_T, these equations uniquely define the unknown switching rates *f* and *r*.

This is how we obtained the "ancestral" values of g_H, g_L, *f*, and *r* for each environmental condition. These values are listed in **Appendix Table S1**. We assumed that Zeocin did not affect the switching rates *f* and *r*.

3.2. Mathematical model of evolutionary dynamics

We now consider a simple deterministic model of how PF cells grow and mutate as a population of haploid asexual cells that are initially clonal (genetically identical). We assume that potentially beneficial mutations enter this ancestral population at a constant overall rate μ, measured per genome per generation. Each incoming mutation is grouped into one of a discrete number of classes denoted *i*, corresponding to mutant populations of size M_i. Each class is characterized by its fitness, f_i. The ancestral genotype's population size is M_0 and its fitness is f_0. The probability of an incoming mutation (on the ancestral genotype background) belonging to class $i \neq 0$ is P_i. We assume that the total population size $N = \sum_i M_i$ remains constant in time.

Our goal is to develop a system of ordinary differential equations (ODEs) that describes the population size of each genotype *i* over time. This requires knowing the "gain" and "loss" rates of each genotype as a function of all genotypes present in the population. Therefore, we defined the influx (gain) and outflux (loss) rates for each genotype as follows.

To obtain the influx rate of new genotypes arising from mutations on the ancestral genotype background we assumed that mutations arise strictly due to errors during the genome replication phase of the cell cycle. Therefore, the number of potentially beneficial mutations arising per unit time should be proportional to the number of genome replications (which is equal to the number of cell divisions) per unit time. For simplicity we ignored the possibility of multiple mutations co-occurring in the same genome. To estimate how many cell divisions occur per unit time, we considered the exponential growth law, according to which the rate of new cell birth in a sufficiently short time interval Δt is proportional to the current number of ancestral genotype cells M_0 and their division rate (fitness) f_0:

$$\frac{\Delta M_0}{\Delta t} = f_0 M_0 . \tag{8}$$

Since each of these newborn cells results from a cell division (and requires a genome replication), the rate of genome replications for ancestral genotype cells is also

$$r_0 = f_0 M_0 . \tag{9}$$

If the beneficial mutation rate per genome per generation is μ then the influx of potentially beneficial mutations will be

$$\Phi_0 = \mu f_0 M_0 . \tag{10}$$

Tying mutations to cell division (or DNA replication) events is a biologically reasonable assumption because spontaneous mutations tend to arise through DNA replication errors. This does not capture the effects of irradiation or other DNA damaging agents, which can generate mutations even without replication. Zeocin is in fact a DNA-damaging agent, but the DNA damage it induces stalls replication forks until the damage is repaired – with or without leftover errors. Therefore, we can still consider only mutations tied to DNA replication for sake of simplicity and to keep our assumptions minimal.

The influx term Φ_0 in **Eqn. 10** only accounts for new potentially beneficial genotypes entering the population (a random cell acquiring a mutation). Even though a mutation appears in the population, it can still be lost by genetic drift, especially in small populations (since genetic drift forces are ~1/*N*, where N is the population size). The chance for a given mutation type $i \neq 0$ to survive drift or "establish" is typically given as $p_i(Est) = 2s_i$, in terms of the selection coefficient (Kimura, 1964)

$$s_i = \frac{f_i - F}{F}, \tag{11}$$

where $F = \frac{\sum_j f_j M_j}{\sum_j M_j}$ is the average fitness over all genotypes.

We considered the influx of new genotypes that survive drift, while ignoring all other mutations because they go extinct very rapidly. Hence, the effective influx of genotypes M_i that carry a potentially beneficial mutation of type *i* and survive drift equals:

$$\Phi_0 \times P_i \times p_i(Est) = 2\mu f_0 M_0 P_i \frac{f_i - F}{F}, \tag{12}$$

where P_i, the probability of the incoming mutation to be of type *i*, obeys $\sum_{i \neq 0} P_i = 1$.

Assuming exponential growth for each sub-population genotype and enforcing constant total population size, we obtain a system of ODEs that describes the evolutionary dynamics:

$$\frac{dM_i}{dt} = 2\mu f_0 M_0 P_i \frac{f_i - F}{F} + f_i M_i - F M_i, \qquad \text{for } i \neq 0$$

$$\frac{dM_0}{dt} = -\frac{2\mu f_0 M_0}{F}\sum_{i>0} P_i(f_i - F) + f_0 M_0 - FM_0 \tag{13}$$

We now apply this model specifically to PF cells. Let the three mutant types be *K*, *T*, *G* with fitnesses f_K, f_T, f_G assigned as follows (using parameters from **Appendix Table S1**):

f_K = ancestral genotype population fitness in D0Zx, where Zx indicates the current Zeocin concentration;

f_T = a uniformly distributed number ranging between $[0, f_K]$;

f_G = 0 in DxZ0; otherwise a uniformly distributed value from $[0, f^*]$ where f^* = height of the cellular fitness peak. For each condition, there is a single fitness maximum (see **Fig. 1B**). We chose values from the uniform distribution because it is the most unbiased assumption (any other distribution would imply that certain fitness values are more likely to occur than others).

The system of ODEs for the PF cells is therefore:

$$\begin{aligned}\frac{dM_K}{dt} &= 2\mu f_0 M_0 P_K \frac{f_K(M_0 + M_T + M_G) - (f_0 M_0 + f_T M_T + f_G M_G)}{f_0 M_0 + f_K M_K + f_T M_T + f_G M_G} + f_K M_K - \\ &- M_K \frac{f_0 M_0 + f_K M_K + f_T M_T + f_G M_G}{M_0 + M_K + M_T + M_G}\end{aligned} \tag{14a}$$

$$\begin{aligned}\frac{dM_T}{dt} &= 2\mu f_0 M_0 P_T \frac{f_T(M_0 + M_K + M_G) - (f_0 M_0 + f_K M_K + f_G M_G)}{f_0 M_0 + f_K M_K + f_T M_T + f_G M_G} + f_T M_T - \\ &- M_T \frac{f_0 M_0 + f_K M_K + f_T M_T + f_G M_G}{M_0 + M_K + M_T + M_G}\end{aligned} \tag{14b}$$

$$\begin{aligned}\frac{dM_G}{dt} &= 2\mu f_0 M_0 P_G \frac{f_G(M_0 + M_K + M_T) - (f_0 M_0 + f_K M_K + f_T M_T)}{f_0 M_0 + f_K M_K + f_T M_T + f_G M_G} + f_G M_G - \\ &- M_G \frac{f_0 M_0 + f_K M_K + f_T M_T + f_G M_G}{M_0 + M_K + M_T + M_G}\end{aligned} \tag{14c}$$

$$\begin{aligned}\frac{dM_0}{dt} &= -2\mu f_0 M_0 \frac{(f_K P_K + f_T P_T + f_G P_G)M_0 - f_0(P_K + P_T + P_G)M_0}{f_0 M_0 + f_K M_K + f_T M_T + f_G M_G} - \\ &-2\mu f_0 M_0 \frac{(f_T - f_K)(P_T M_K - P_K M_T) + (f_G - f_K)(P_G M_K - P_K M_G) + (f_G - f_T)(P_G M_T - P_T M_G)}{f_0 M_0 + f_K M_K + f_T M_T + f_G M_G} + \\ &+ f_0 M_0 - M_0 \frac{f_0 M_0 + f_K M_K + f_T M_T + f_G M_G}{M_0 + M_K + M_T + M_G}\end{aligned} \tag{14d}$$

We solve this ODE system numerically, scanning the mutation rate μ and the mutation type probabilities P_K and P_T to predict the population structure [M_0, M_K, M_T, M_G] and half-life of the ancestral genotype in **Appendix Figure S2**.

3.3. Evolutionary simulation framework

We developed a more detailed simulation method to predict the population dynamics of mutations arising randomly in periodically resuspended, liquid cell cultures. The framework is a hybrid between the deterministic population dynamics model presented in **Eqn. 7a** above, which captured switching rates between high and low PF gene circuit expressors as well as their condition-dependent growth rates, and a Poisson process producing mutant alleles from the ancestral genotype population (**Appendix Figure S1C**). The purpose of this framework is to predict the range of possible evolutionary dynamics by representing what we believe to be the most important factors dictating the initial evolutionary dynamics of PF-carrying populations in conditions with or without inducer (Doxycycline) and antibiotic stress (Zeocin). Most parameters used in the framework were estimated from our characterization of the fitness landscape (**Fig. 1B**) or in previous work (Nevozhay et al, 2012). Overall, we were left with four free parameters that are scanned as outlined below: beneficial mutation rates with and without Zeocin (μ_{+Z}; μ_{-Z}), and probabilities of the types of mutations that can arise as outlined in the main text. Finally, using the scans we were able to select values for the free parameters that offer excellent agreement with our evolution experiments.

We approximate each yeast cell carrying the PF synthetic gene circuit by a two-state model of growth and phenotypic switching as described above (**Eqn. 7**). Therefore, the mean time (in generations) a cell remains in a given expression state is τ_L=ln(2)/r for L cells and τ_H=ln(2)/f for H cells, which we can interpret as the "memory" of that state. We assumed that the switching rates r and f depend on the Doxycycline concentration, while the growth rates g_H and g_L depend on the concentrations of both Doxycycline and Zeocin. Next, we outline the evolutionary simulation algorithm (implemented in Python 3.4), followed by a description of each step in the algorithm.

We begin each simulation with a single population of 10^6 cells all with the ancestral genotype and in the L expression state, similar to the experiments. All parameters g_L, g_H, r, and f for the ancestral genotype (**Appendix Table S1**) are obtained by fitting as described above, using experimental fitness and gene expression measurements in 13 different Doxycycline and Zeocin concentrations (**Fig. 1B, Appendix Figure S1A**).

To simulate potentially beneficial mutant alleles as they arise, we randomly generated and introduced them into the population as a Poisson process with an overall rate of μ (/genome /generation). Each simulated mutant is described by the parameter set [g_{Lp}, g_{Hp}, r_p, f_p], where the subscript "p" (for "perturbation") indicates that the parameter might differ from its ancestral value. Mutants can be classified into one of three types based on their parameter set. The three mutation classes are:

1. K type or "Knockout" mutations: [g_{Lp}, g_{Hp}, r_p, f_p]=[g_L, g_L, 0, g_H]. They correspond to complete knockout of rtTA function. We assumed both H and L cells of K mutants to grow at rate g_L.
2. T type or "Tweaking" mutations: [g_{Lp}, g_{Hp}, r_p, f_p]=[g_L, g_{Hp}, $r_p(g_{Hp})$, $f_p(g_{Hp})$]. These mutations affect the induction dynamics of the PF gene circuit without destroying it, jointly altering the parameters g_H, r, and f. Considering that rtTA mutations affect both the growth rate and stability of the H expression state, a random change from g_H to g_{Hp} should impose associated changes from r to r_p and from f to f_p. Therefore, we modeled the relationship between τ_{Lp}, τ_{Hp} and g_{Hp} assuming that all of these variables depend on the Doxycycline concentration D.

For simplicity, we postulated that g_{Hp} and the Doxycycline concentration D are linearly related: $D = a\, g_{Hp} + b$, which is equivalent to $g_{Hp} = (D - b)/a$. There are two free parameters in this relationship. We can determine them on the grounds that the relationship must satisfy two constraints. First, at the maximum concentration D_{max} (e.g., if cells evolve in D2Z0, D_{max} = [Dox] = 2 μg/ml), we should have $g_{Hp} = g_H$ (ancestral growth rate). Second, the maximum possible growth rate should be attained at minimum effective amount of Doxycycline to which the circuit is sensitive, D_{min}, defined by $D_{min} = D_{max}(1 - g_{Hmax}/g_0)$: $g_{Hmax} = g_{Hp}(D_{min})$. Solving these two equations allows us to obtain a and b in terms of D_{min}, D_{max}, g_H, and g_{Hmax}. Thus we obtain the slope a=-(D_{max}-D_{min})/(g_{Hmax}-g_H) and the intercept $b = D_{max} - a g_H$. In expanded form the linear mapping between g_{Hp} and D is:

$$D = \frac{D_{max} - D_{min}}{g_{Hmax} - g_H}(g_H - g_{Hp}) + D_{max} \quad (15)$$

The value D also defines the half-lives $\tau_L = log(2)/r$ and $\tau_H = log(2)/f$ of the L and H states. We applied a phenomenological model to interpolate the relationship between τ_{Lp} and τ_{Hp} and inducer concentration D extracted from experimental data (**Fig. 1**; **Appendix Figure S3E**):

$$\begin{aligned} \tau_{Hp} &= a_0 D + a_1 D^2 \\ \tau_{Lp} &= a_2 D^{-1} + a_3 \end{aligned} \quad (16)$$

where a_0, a_1, a_2, and a_3 are constants obtained from fitting **Eqn. 16** to the experimental data. Finally, we end up with perturbed parameters $r_p = \log(2)/\left(a_2 D^{-1} + a_3\right)$ and $f_p = \log(2)/\left(a_0 D + a_1 D^2\right)$. In summary, these constraints ensure that a change from g_H to g_{Hp} also lowers the mean expression level by altering switching rates (since all are rtTA-mediated). The relevant parameter values are given in **Appendix Table S1**.

3. G type or "Generic" mutations: [g_{Lp}, g_{Hp}, r_p, f_p]=[g_{Lp}, g_{Hp}, r, f]. These are extra-rtTA mutations affecting g_L and g_H, slightly improving drug resistance independently of rtTA. We scale the fitness improvements of g_{Lp} and g_{Hp} such that they have the same percent improvement toward g_0, lower in absolute terms for g_H. Specifically, for growth rate change from g_L to g_{Lp}, we have:

   $g_{Hp} = g_H\,(g_{Lp}/g_L)\,(g_0 - g_H)/g_0$.

   Here, the factor g_{Lp}/g_L scales g_H by the level of fitness increase affecting L cells. If the fitness of H cells would increase proportionally, it would equal $g'_{Hp} = g_H\left(g_{Lp} / g_L\right)$. The factor $(g_0 - g_H)/g_0$ <1 then lowers g'_{Hp}, in a manner proportional to how far g_H is from the fitness peak g_0.

Additionally, the three probabilities required for implementing the occurrence of mutations in these simulations are: μ, which defines the rate of potentially beneficial mutations, and two out of P(T), P(K), and P(G), which define the likelihood of each mutation type. In conditions lacking Doxycycline, the population is unimodal (r = 0), thus the only beneficial mutation type is G.

To determine fixation probabilities for new alleles, we need to account for two effects: the probability of extinction of slightly favorable alleles by drift, and the probability of allele loss during resuspension.

The probability of a slightly favorable mutation fixing is approximately 2*s*, where *s* is the selection coefficient of the mutant strain (Kimura, 1964). The chance of surviving resuspensions (periodic bottlenecks) has been treated before (Wahl & Gerrish, 2001). Using this earlier model with our experimental populations and resuspension frequency, we found that the chance of losing newly generated beneficial alleles is switch-like, with virtually no chance of losing a new allele by resuspensions if it arose in the first four hours after the previous resuspension, and with a very high probability of losing it thereafter. Our simulation framework used these two factors to determine the chance of a newly generated allele *i* surviving at a time *t* hours post-resuspension: $p_i(t) = 2s\theta(4-t)$ where θ is the Heaviside step function.

Considering that the total number of cells is a continuous variable in these simulations, we set the initial number of mutant cells to represent 1 in the culture assuming a population size determined by 12 hour resuspensions (cultures starting with ~10^6 cells). Mutant genotypes arising in a given simulation run all derive from the ancestral genome. For simplicity, we do not allow multiple mutations to occur in the same genome.

In the beginning of the simulations, when cells are first exposed to the environment in which they evolve, they undergo transient dynamics as Doxycycline and Zeocin diffuse into the cells and induce the PF gene circuit. We capture the transient effects of Doxycycline by the dynamics of the built-in two-state model (all cells starting as *L*, and then some switching to *H*). To capture the known delay arising as Zeocin crosses the cell and nuclear membranes and binds to DNA before affecting fitness, we use the internalization dynamics model from **Eqn. 2**, and re-estimate Zeocin internalization parameters based on our experimentally observed initial fitness dynamics in D0Z2, as detailed below. Using this algorithm, we predict dynamics of overall population fitness, populations of mutant strains, distributions of mutation classes, and timing of mutations (**Figs. 2;3F-G;4C;5F-G; Appendix Figure S2**).

3.4. Steps in the algorithm

1. **Initialization**. Assume 10^6 cells (all in state L, corresponding to the condition D0Z0). Select parameter set appropriate for environment. For Zeocin-containing environments, use a time-dependent growth rate parameter $g_L = g_0\,\gamma_Z(t)$ where g_0 is the initial fitness (in D0Z0) and $\gamma_Z(t)$ is the time-dependent fitness effect of Zeocin (Nevozhay et al, 2012). This captures the dynamics of growth rate relaxation to a lower steady state value as Zeocin, Z_i, enters the cells. This corresponds to the solution of **Eqn. 2a** above, assuming no PF circuit activity initially (R = 0):

$$\gamma_Z(t) = \frac{\chi}{Z_i(t)+\chi}$$
$$Z_i(t) = \frac{Z\phi}{h_z}\left(1-e^{-h_z t}\right) \qquad (17)$$

2. **Calculate time-dependent ancestral genotype PF population size**. The total number of PF cells with intact (ancestral) genotype is: $N_{tot,PF}(t) = N_L(t) + N_H(t)$. More explicitly, with $N_H(0) = 0$ we have the time-dependent solution of **Eqn. 7a** above:

$$N_{tot}(t) = \frac{1}{2\lambda} N_L(0) e^{\omega t}\left(g_H - f - r - g_L + \lambda + e^{\lambda t}\left(f - g_H + r + g_L + \lambda\right)\right)$$
$$\omega = -\frac{1}{2}\left(f - g_H + r - g_L + \lambda\right) \qquad (18)$$
$$\lambda = \sqrt{f^2 + \left(g_H + r - g_L\right)^2 + 2f\left(r - g_H + g_L\right)}$$

3. **Generate next mutation time**. For $t \in [0, 480]$ hours, calculate the next mutation time using the effective beneficial mutation rate, μ. For a Poisson process with mutation i at time t_i, the next mutation $i+1$ occurs at time t_{i+1} at instantaneous rate $\frac{\mu w \mathrm{N}_{\mathrm{tot,PF}}(t_i)}{\log(2)}$, where w is the overall population fitness at time t_i and the factor $w/\log(2)$ converts the units of μ from 1/generations to 1/hours. This model is approximately correct as long as the mutation rate is sufficiently high, because then the time between mutations is short enough that the population is approximately constant between mutation events. We thus draw the mutation time t_{i+1} from the probability distribution $\mathrm{P}(t_{i+1}) = \frac{\mu w \mathrm{N}_{\mathrm{tot,PF}}(t_i)}{\log(2)} e^{-\frac{\mu w \mathrm{N}_{\mathrm{tot,PF}}(t_i)}{\log(2)} t_{i+1}}$.
The mutation is retained in the population only if it occurs within the first four hours after a multiple of the resuspension time (12 hours) as explained in the previous section.
4. **Select the mutation type**. Let $\xi(a,b)$ be a uniformly distributed random real number in the range $[a,b]$. Each mutation event is randomly assigned to one of three classes T, K, or G with probability P(T), P(K), and P(G), respectively, implementing the perturbations depicted in **Appendix Figure S1B** (see section 3.3 above):

•"Tweak" T

$$g_{Hp} = \xi(g_H, g_{Hmax});\ r_p = \log(2)\big/\left(a_2 D^{-1} + a_3\right);\ f_p = \log(2)\big/\left(a_0 D + a_1 D^2\right) \quad \text{(19a)}$$

•"Knockout" K

$$r_p = 0;\ f_P = g_H;\ g_{Hp} = g_L \quad \text{(19b)}$$

•"Generic" G

$$g_{Lp} = g_L\,[1 + \xi(0, \varphi)];\ g_{Hp} = g_H\,(g_{Lp}/g_L)\,(g_0 - g_H)/g_0 \quad \text{(19c)}$$

where φ reflects the height of the fitness peak relative to the current population fitness (roughly, the slope of the fitness landscape) in each condition. The value of φ gives the maximal attainable fitness by a G-type mutation. Its value is not critical as long as it does not allow mutations with unrealistically exaggerated benefits, while at the same time ensuring the maximal benefit without Zeocin is small. We therefore chose $\varphi = 0.8$ in Zeocin-containing conditions and $\varphi = 0.01$ in Zeocin-free conditions.

5. **Define the initial size and state of each mutant population**. When a new mutant arises after the first resuspension, we need to scale its initial population to reflect what its population would be in culture assuming that new mutants start as a single cell. We call this population M_{ic}. Numerically, we allow the population to continue growing at exponential phase for the 20 simulated days, without directly implementing resuspensions in the growing population. Therefore, the fraction of the population representing one cell increases depending on how many 12-hour blocks of time have passed. With 12-hour resuspensions: $M_{ic}(t_m) = \frac{1}{N_{tot}(0)} \sum_{i \in A} N_{tot,i}\left(12\lfloor t_m/12 \rfloor\right)$ where $\lfloor \circ \rfloor$ denotes the floor function, A_i is the quantity of allele i in the population, and $N_{tot}(t)$ reflects total population at time t. Each initial mutant cell is randomly selected to be in the L or H state with probability weighted by fraction of H or L cells in the intact PF population. Note that we still account for population size effects from resuspension bottlenecks, as described in Section 3.3.
6. **Continue the simulation**. Repeat steps 1 – 5 until the end time is reached.
7. **Generate dynamics**. Iterate through the 20 day simulation time in uniform steps, computing allele frequencies for the population.

3.5. Justification of parameter choices

To obtain a realistic value for the effective beneficial mutation rate μ, we need to consider the set of all deletions, base-pair substitutions, frameshifts, etc. that could possibly improve growth rates for each of the conditions where PF cells evolved. This is difficult to estimate, but an upper limit is given by the total mutation rate estimate of 3.80×10^{-10} – 6.44×10^{-10} /bp /generation (Lang & Murray, 2008). Considering the yeast genome size of approximately 10^7 bp, the beneficial mutation rate is probably a few orders of magnitude less than the overall mutation rate of 10^{-3} /genome /generation. Recently the rate of beneficial mutation was experimentally estimated at approximately 10^{-6} /genome /generation (Levy et al, 2015).

For a randomly placed beneficial mutation, what would be realistic choices for P(T), P(K), or P(G)? Because P(T) + P(K) + P(G) = 1, we only need to have estimates for two of the probabilities. On the one hand, the overall chance of extracircuit beneficial mutation may be much higher than intracircuit because of the relative quantities of genetic material. However, considering that the PF gene circuit strongly affects fitness in most environments where the cells evolve, beneficial intracircuit mutations may be more likely to establish than extracircuit mutations in the early phase of evolution. Therefore, we scanned these probabilities over 3 orders of magnitude in the simulations.

4. Extended results of evolutionary dynamics simulations

4.1. The effects of various parameters on evolutionary dynamics

To study the effects of various mutational regimes on the first adaptive step in evolution, we defined two quantitative, experimentally meaningful, measures. First, we calculated the number of mutant alleles reaching a frequency > 5% (our threshold for detection with whole-genome Illumina sequencing). Second, we determined the ancestral strain's half-life over 20 days of simulated time for a range of effective beneficial mutation rates less than 10^{-5} /genome /generation, simultaneously scanning the probabilities P(T) and P(G) (**Appendix Figure S3**).

In the condition with the least selective pressure, DiZ0, none of the mutants reached a very high frequency by Day 20. Therefore, the simulations predicted uniformly long ancestral half-life, regardless of the overall beneficial mutation rate (**Appendix Figure S3A,B**).

With higher Doxycycline (D2Z0), more mutant alleles could establish to levels >5% in 20 days, and the average ancestral genome half-life (the time for the ancestral genome frequency to reach 50% of the population) was the shortest among all conditions (**Appendix Figure S3A,B**). The beneficial mutation rate μ affects the stability of the ancestral strain (i.e. its genome half-life) most strongly at very low values of μ, where the ancestral genotype's half-life falls from nearly the maximum measurable (20 days) to around 5 days (**Appendix Figure S3B**) in the same parameter regime where the number of alleles is low. This indicates that having one or just a few mutants that improve fitness is sufficient for a fast sweep in this condition. At the same time, for $\mu > 10^{-6.5}$/genome /generation, the ancestral genotype half-life decreases less dramatically with the mutation rate (**Appendix Figure S3B**). In this regime many mutations establish, each with higher fitness than the ancestral genotype. The non-monotonic relationship between number of alleles and mutation rate in this condition reflects a large number of alleles establishing at levels below 5 percent taking up part of the population at higher mutation rates.

When both Doxycycline and Zeocin are present (DiZ2 and D2Z2), increasing μ results in a higher number of established mutants (**Appendix Figure S3A**). The number of mutants increases with P(T), while the half-life of the ancestral genotype decreases with P(T) (**Appendix Figure S3B**). The stronger

selective pressure in D2Z2 results in shorter ancestral genotype half-life than DiZ2 for most parameter values.

The effect of P(G) on the number of mutants and ancestral half-life is small for much of the parameter space (different line thicknesses reflect P(G) = 5%, 75%, and 95% for a given P(T) in **Appendix Figure S3A,B**). One exception to this is trend is D0Z2, for which the ancestral genome's half-life decreases, and the mutant allele count somewhat increases, with P(G) (**Appendix Figure S3A,B**).

Based on the relative number of total intra- and extra-circuit mutations observed in the experiments with Zeocin and simulation results reflecting them (**Fig. 2; Appendix Figure S3**) we set P(G) = 0.75 (i.e. 75 %). Probabilities P(T) and P(K) were then estimated by matching the timing and total number of emergent mutations to experimental values; see the main text. Based on that analysis, we chose the values P(T|¬G) = P*(T) = 10% and P(K|¬G) = P*(K) = 90%, respectively (out of total PF-only mutations). This translated into probability values of P(T) = 2.5% and P(K) = 22.5%, the rest being generic (G-type) mutations.

4.2. Agreement between predicted and observed evolutionary dynamics

We used our calibrated simulation framework to explore the evolutionary dynamics in representative cases (**Figs. 3F,G;4C;5F,G; Appendix Figure S3D,E**). In each simulated case, the fitness transiently drops to a level dependent on the environment (i.e. quantity of Doxycycline and absence or presence of Zeocin). Then the population begins to recover at a rate proportional to the probability and benefit of mutations in that condition (**Appendix Figure S3D**). In D2Z0, fitness drops in individual simulations for the first few days, and then recovers coincidently with K alleles sweeping the population (compare **Appendix Figure S3D** and **Fig. 3F**). In DiZ0 fitness drops slightly and then recovers minimally because selection is too weak (compare **Appendix Figure S3D** and **Fig. 3G**). In D2Z2 and DiZ2 fitness drops similarly and then recovers coincidently with the rise of fitter mutant alleles (compare **Appendix Figure S3D** and **Figs. 5F,G**).

We next reconstructed time courses of mean PF gene expression (corresponding to both rtTA and ZeoR::yEGFP concentration) for the evolving, genotypically mixed population. We used cellular fitness landscapes to infer expression levels (see above). Mean expression levels initially increase, then decrease, in each condition (**Appendix Figure S3F**), except for D0Z2 and D0Z0. K mutations disable PF, causing a clear rise and then fast fall of gene expression in D2Z0 (dark blue line, **Appendix Figure S3F**). In DiZ0 gene expression drops only slightly, reflecting low selection (light blue line, **Appendix Figure S3F**). The reconstructed expression levels in DiZ2 and D2Z2 also capture the experimentally measured trends (green and magenta lines, **Appendix Figure S3F**).

5. Appendix References

DePristo MA, Banks E, Poplin R, Garimella KV, Maguire JR, Hartl C, Philippakis AA, del Angel G, Rivas MA, Hanna M, McKenna A, Fennell TJ, Kernytsky AM, Sivachenko AY, Cibulskis K, Gabriel SB, Altshuler D, Daly MJ (2011) A framework for variation discovery and genotyping using next-generation DNA sequencing data. *Nat Genet* **43:** 491-498

Garrison E, Marth G (2012) Haplotype-based variant detection from short-read sequencing. *arXiv* **q-bio.GN:** 1207.3907

Kimura M (1964) Diffusion models in population genetics. *J Appl Prob* **1:** 177-232

Lang GI, Murray AW (2008) Estimating the per-base-pair mutation rate in the yeast Saccharomyces cerevisiae. *Genetics* **178:** 67-82

Langmead B, Salzberg SL (2012) Fast gapped-read alignment with Bowtie 2. *Nat Methods* **9:** 357-U354

Levy SF, Blundell JR, Venkataram S, Petrov DA, Fisher DS, Sherlock G (2015) Quantitative evolutionary dynamics using high-resolution lineage tracking. *Nature* **519:** 181-186

Li H (2013) Aligning sequence reads, clone sequences, and assembly contigs with BWA-MEM. *arXiv* **q-bio.GN:** 1303.3997v1302

Li H, Handsaker B, Wysoker A, Fennell T, Ruan J, Homer N, Marth G, Abecasis G, Durbin R, Proc GPD (2009) The Sequence Alignment/Map format and SAMtools. *Bioinformatics* **25:** 2078-2079

McKenna A, Hanna M, Banks E, Sivachenko A, Cibulskis K, Kernytsky A, Garimella K, Altshuler D, Gabriel S, Daly M, DePristo MA (2010) The Genome Analysis Toolkit: A MapReduce framework for analyzing next-generation DNA sequencing data. *Genome Res* **20:** 1297-1303

Robinson JT, Thorvaldsdottir H, Winckler W, Guttman M, Lander ES, Getz G, Mesirov JP (2011) Integrative genomics viewer. *Nat Biotechnol* **29:** 24-26

Thorvaldsdottir H, Robinson JT, Mesirov JP (2013) Integrative Genomics Viewer (IGV): high-performance genomics data visualization and exploration. *Brief Bioinform* **14:** 178-192

Wahl LM, Gerrish PJ (2001) The probability that beneficial mutations are lost in populations with periodic bottlenecks. *Evolution* **55:** 2606-2610

6. Appendix Figure Legends

Appendix Figure S1. Fitness landscape and predicted mutations. (A) The population fitness landscape in **Fig. 1B** was fit to experimental data points in various combinations of Doxycycline and Zeocin doses. There are N = 3 replicates per environmental condition in the fitness landscape. (B) Expected mutation types that could arise in the landscape and their effects on population dynamics: ancestral genotype (PF), knockout mutations (K), tweaking mutations (T), and extra-rtTA mutations (G). Red arrows denote processes altered by each mutation type. (C) Simplified flowchart for the simulation framework provides an overview of the steps involved in predicting evolutionary dynamics.

Appendix Figure S2. Predicted environment-dependent characteristics of evolutionary dynamics. Shown are the fraction of genotypes at 20 days and half-life of ancestral genotype from the ODE model as a function of all free parameters.

(A) Calculated (predicted) fraction of wild-type (W), and K, T, and G type mutants at day 20 in DiZ0.

(B) Calculated (predicted) half-life of the ancestral genotype in DiZ0.

(C) Calculated (predicted) fraction of wild-type (W), and K, T, and G type mutants at day 20 in D2Z0.

(D) Calculated (predicted) half-life of the ancestral genotype in D2Z0.

(E) Calculated (predicted) fraction of wild-type (W), and K, T, and G type mutants at day 20 in D0Z2.

(F) Calculated (predicted) half-life of the ancestral genotype in D0Z2.

(G) Calculated (predicted) fraction of wild-type (W), and K, T, and G type mutants at day 20 in D2Z2.

(H) Calculated (predicted) half-life of the ancestral genotype in D2Z2.

(I) Calculated (predicted) fraction of wild-type (W), and K, T, and G type mutants at day 20 in DiZ2.

(J) Calculated (predicted) half-life of the ancestral genotype in DiZ2.

Appendix Figure S3. Predicted evolutionary dynamics from the simulation framework.

(A, B) Predicted effects of mutation rates on characteristics of evolutionary dynamics (number of alleles >5% of the population and half-life of the ancestral genome) in each environmental condition from **Fig. 1B**; see the legend in panel D for colors in all panels. Multiple lines of the same color but different thickness depict a scan of extra-rtTA (type G) mutation probabilities: $P(G)$=5% (thin), $P(G)$=75% (medium), and $P(G)$=95% (thick). For intra-rtTA mutations, we give the probabilities P(T) and P(K) as fractions of total intra-rtTA mutations: P*(T) = $\mathrm{P}\left(\mathrm{T}\middle|\neg\mathrm{G}\right)$ = P(T) / (1 - P(G)) and P*(K) = $\mathrm{P}\left(\mathrm{K}\middle|\neg\mathrm{G}\right)$ = P(K) / (1 - P(G)). This way we can consider the relative frequencies of intra-rtTA mutations independently of the probability of G mutations. We ran 20 simulations while scanning the effective beneficial mutation rate, μ. Error bars report mean ± SEM.

(C) Fraction of G type mutants established in DiZ2 versus $P(T)$ and $P(G)$, with $\mu_{+Z} = 10^{-5.4}$. For these parameter scans, we ran 50 simulations per point in parameter space.

(D) Sample fitness timecourses for each of the conditions, corresponding to the simulated allele frequency graphs shown in **Figs. 3-5**. For these results we ran 500 simulations per condition.

(E) Half-lives of low (τ_L) and high (τ_H) expression states of PF cells versus Doxycycline doses, estimated from experimental data. Lines represent phenomenological models given in **Eqn. 16**.

(F) Predicted fluorescence levels back-calculated from fitness landscapes (**Fig. 1B**). To calculate the fluorescence for each condition, we used the cellular fitness of high and low expressors to infer their subpopulation sizes and then estimated the mixed population-average fluorescence as a weighted mean over the subpopulations for each environment at each time point.

Appendix Figure S4. Time courses and phenotypes for evolution in D2Z0.

(A) Experimentally measured fluorescence and fitness (3 replicates) for 24 h resuspensions of PF cells in D2Z0.

(B) Allele frequency time courses from a 24 h resuspension experiment (replicate #1) in Doxycycline alone (D2Z0).

(C) Gene expression shift of clonal isolate #1 compared to the PF ancestor in condition D2Z0, 24-hour resuspensions. Clone #1 is unresponsive to inducer. All other 5 clones tested behave identically.

(D) Coverage profile of reads aligned to the wild-type reference genome (top) and a reference with the 30-bp duplication included (bottom) for a frozen sample taken at Day 19 from 12hr-D2Z0-r1. Horizontal darker bar covers original 30 bp region on top and the new 60 bp region on the bottom.

(E) Coverage profile of reads aligned to the wild-type reference genome (top) and a reference with the 78-bp deletion included (bottom) for a frozen sample taken at Day 19 from 24hr-D2Z0-r3. Horizontal darker bar covers the original 78 bp region on top, which is deleted on the bottom.

Appendix Figure S5. Time courses and phenotypes for evolution in D0Z2.

(A) Experimentally measured fluorescence and fitness (3 replicates) for 24 h resuspensions of PF cells in D0Z2.

(B) Time course of allele frequencies inferred directly from whole-genome sequencing of frozen samples in D0Z2 experiment with 24 h resuspensions, replicate #1. Sanger sequencing showed linkage between some alleles that whole-genome sequencing could not detect (**Fig. 4B**).

(C) Two control strains (PX and NR) and their basal expression levels compared to PF cells. PX lacks yEGFP:ZeoR, while NR is a different gene circuit with higher basal expression of yEGFP:ZeoR.

(D) Fitness of PX versus PF cells in D0Z2 over several days. Cells without yEGFP:ZeoR vanish after 1 day in D0Z2.

(E) Growth curve of NR versus PF cells in various Zeocin concentrations. NR resists Zeocin better due to its higher basal expression.

Appendix Figure S6. Time courses and phenotypes for evolution in D2Z2.

(A) Experimentally measured fluorescence and fitness (3 replicates) for 24 h resuspensions of PF cells with 24 h resuspensions in Doxycycline inducer and Zeocin antibiotic stress (D2Z2).

(B) Time course of allele frequencies inferred from next-generation and Sanger sequencing of frozen samples in environment D2Z2 with 24 hour resuspensions, replicate #1.

(C) Tradeoffs between fitness in the presence and absence of Zeocin for the ancestral PF gene circuit and clones evolved in D2Z2.

(D) Experimentally measured fluorescence and fitness replicates for resuspensions of PF cells in media containing neither Doxycycline inducer nor Zeocin (D0Z0) with 12-hour resuspensions.

(E) Experimentally measured fluorescence and fitness replicates for resuspensions of PF cells in media containing neither Doxycycline inducer nor Zeocin (D0Z0) with 24-hour resuspensions.

Appendix Figure S7. Phenotyping of clones and reconstructed mutants.

(A) Gene expression distributions of clonal isolate #1 from DiZ2 in various conditions.

(B) Phenotypes of mutations reconstructed in the ancestral background. Mutations are from clones #1 and #2 evolved in Doxycycline and Aeocin (D2Z2, "suboptimal response"). Top: log10-ratio of fitness with Doxycycline (D2Zy) relative to no Doxycycline (D0Zy) either with or without Zeocin (y=0 or y=2). Middle: log10-ratio of average fluorescence intensity with Doxycycline (D2Zy) relative to no Doxycycline (D0Zy). Bottom: log10-ratio of fitness with Zeocin (DxZ2) relative to no Zeocin (DxZ0), either with or without Doxycycline (x=0 or x=2). Error bars represent standard deviations around the mean. Stars denote significance at $P<0.05$.

(C) Gene expression of clonal isolate #1 and the corresponding reconstructed strain (dashed line) from D2Z2 in various conditions.

(D) Gene expression of clonal isolate #2 and the corresponding reconstructed strain (dashed line) from D2Z2 in various conditions.

Appendix Figure S8. Predicted frequencies of pre-existing mutations in the simulation framework. To simulate the effect of pre-existing neutral mutations on evolutionary dynamics, we performed a simulation with neither Doxycycline nor Zeocin (D0Z0) for 24h. We saved all of the neutral PF mutations that arose, and then simulated further evolution in one of the five Doxycycline- and/or Zeocin-containing conditions (D2Z0, DiZ0, D0Z2, D2Z2, DiZ2). (A) Final allele frequency (at Day 20) of mutants that arose neutrally in the initial 24h simulation time. Error bars represent the SEM. (B) Comparison between simulations with one day of neutral mutation accumulation (P, "preexisting") and without it (A, "purely ancestral") reveals nearly identical mean and standard deviation of the ancestral genotype's half-life in all conditions. Error bars: standard deviation. $N = 500$ runs.

7. Appendix Tables

Param	Value					Notes
μ_{+Z}	$10^{-5.4}$ /genome /generation					Mut. rate in Zeocin.
μ_{-Z}	$10^{-6.2}$ /genome /generation					Mut. rate without Zeocin.
h_z	0.1 /h					Zeocin outflux rate.
Z	2 mg/ml					Zeocin concentration.
χ	0.01076 mg/ml					$\chi = \phi Z/h_z$; Zeocin-DNA assoc.
s	186.7983 ml/(h mg)					Zeocin-ZeoR association rate.
ϕ	5.38×10^{-4} /h					Zeocin internalization rate.
α	0.2229 mg/ml					rtTA toxicity.
β	0.4424 mg/ml					Dox - rtTA association.
g_0	0.2634 /h					Ancestral growth rate in D0Z0.
g_{Li}	g_0					Initial growth rate in Zeocin.
a_0 (τ_H)	1091.07					Params. for curves in **Appendix Figure S2E**.
a_1 (τ_H)	320.901					
a_2 (τ_L)	14.1197					
a_3 (τ_L)	-1.28361					
	**DiZ0**	**DiZ2**	**D2Z0**	**D2Z2**	**D0Z2**	Condition-specific rates. /h
g_L	g_0	0.50 g_0	g_0	0.50 g_0	0.50 g_0	Low expressor growth rate.
g_H	0.75 g_0	0.75 g_0	0.60 g_0	0.60 g_0	N/A	High expressor growth rate.
r	0.01	0.01	0.12	0.12	0.0000	L → H switch rate.
f	0.003	0.003	0.0002	0.0002	N/A	H → L switch rate.
g_{Hmax}	g_0	g_0	g_0	0.9 g_0	g_0	Maximum possible g_H.

Appendix Table S1. Parameters used in mathematical and computational models of PF gene circuit evolution.

Experiment D0Z0	Location	Mutation	Read counts	
			**Day 1**	**Day 19**
12hr, r1	*RAD1* 1350<br>(chr 16: 508,046)	A → C<br>(Synonymous)	Illumina: 9% (6/68)<br>Sanger: NA	Illumina: 14% (5/37)<br>Sanger: NA

Appendix Table S2. Mutations detected by whole-genome Illumina sequencing and targeted Sanger sequencing in condition D0Z0 (galactose-only). *In all subsequent mutation tables (Appendix* ***Tables S3-S5****) the first column indicates the experiment (12 hr or 24 hr resuspension, and replicate number). The second column indicates the genomic location; we list the nearest feature and the 1-based coordinate relative to that feature (i.e., coordinate 1 is the first base in the gene, while -1 is the last base before the gene, both in the frame of transcription), as well as the chromosomal coordinate (Watson strand) with respect to the S288c reference. The third column lists the effect of the mutation (base changes are with respect to the Watson strand). The remaining columns show the read counts at various days in the experiment. "NA" indicates that sequencing method was not performed at that day, while a gray box indicates neither sequencing method was performed. An asterisk indicates that the mutation did not pass filters, but we report it here for completeness.*

(A) Experiment DiZ0	Location	Mutation	Read counts		
			Day 9	Day 13	Day 19
12hr, r1	rtTA -212 (chr 15: 729,394)	42 bp deletion	Illumina: 0% Sanger: NA	Illumina: NA Sanger: 0%	Illumina: 0% Sanger: 58% (7/12)
12hr, r2	Illumina: NA Sanger: None				
12hr, r3	Illumina: NA Sanger: None				

(B) Experiment D2Z0	Location	Mutation	Read counts	
			Day 9	Day 19
12hr, r1	rtTA 123 (chr 15: 729,728)	G → T (Leu → Phe)	Illumina: 46% (45/97) Sanger: 50% (6/12)	Illumina: 1% (1/69)* Sanger: 0%
	rtTA 153 (chr 15: 729,758)	G → C (Leu → Phe)	Illumina: 11% (12/108) Sanger: 25% (3/12)	Illumina: 0% Sanger: 0%
	rtTA 196 (chr 15: 729,801)	C → G (His → Asp)	Illumina: 11% (13/121) Sanger: 8% (1/12)	Illumina: 98% (58/59) Sanger: 100% (12/12)
	rtTA 410 (chr 15: 730,015)	T → G (Val → Gly)	Illumina: 13% (15/120) Sanger: 8% (1/12)	Illumina: 0% Sanger: 0%
12hr, r2	rtTA 189 (chr 15: 729,796)	C → G (His → Gln)		Illumina: NA Sanger: 40% (4/10)
	rtTA 514 (chr 15: 730,121)	C → T (Gln → stop)		Illumina: NA Sanger: 60% (6/10)
12hr, r3	rtTA 77 (chr 15: 729,684)	C → G (Thr → Arg)		Illumina: NA Sanger: 10% (1/10)
	rtTA 129 (chr 15: 729,736)	G → C (Trp → Cys)		Illumina: NA Sanger: 30% (3/10)
	rtTA 145 (chr 15: 729,752)	C → T (Arg → Trp)		Illumina: NA Sanger: 50% (5/10)
	rtTA 577 (chr 15: 730,182)	3 bp deletion		Illumina: NA Sanger: 10% (1/10)

			Day 2	Day 5	Day 8	Day 9	Day 10	Day 19
24hr, r1	rtTA 91 (chr 15: 729,696)	G → C (Ala → Pro)	Illumina: <1% (1/146)* Sanger: NA	Illumina: 5% (6/116) Sanger: NA	Illumina: NA Sanger: 10% (1/10)	Illumina: NA Sanger: 10% (1/10)	Illumina: 4% (6/161)* Sanger: NA	Illumina: 2% (2/85)* Sanger: 0%
	rtTA 95 (chr 15: 729,700)	30 bp tandem duplication	Illumina: 0% Sanger: NA	Illumina: 0% Sanger: NA	Illumina: NA Sanger: 60% (6/10)	Illumina: NA Sanger: 10% (1/10)	Illumina: 0% Sanger: NA	Illumina: 0% Sanger: 58% (7/12)
	rtTA 454 (chr 15: 730,059)	C → T (Gln → stop)	Illumina: 0% Sanger: NA	Illumina: 0% Sanger: NA	Illumina: NA Sanger: 10% (1/10)	Illumina: NA Sanger: 0%	Illumina: <1% (1/191)* Sanger: NA	Illumina: 2% (1/57)* Sanger: 0%
	rtTA 609 (chr 15: 730,214)	T → A (Cys → stop)	Illumina: 1% (2/134)* Sanger: NA	Illumina: 52% (63/121) Sanger: NA	Illumina: NA Sanger: 20% (2/10)	Illumina: NA Sanger: 20% (2/10)	Illumina: 50% (83/167) Sanger: NA	Illumina: 40% (20/50) Sanger: 42% (5/12)
24hr, r2	rtTA 179 (chr 15: 729,784)	T → C (Leu → Ser)						Illumina: 4% (3/75)* Sanger: 10% (1/10)
	rtTA 442 (chr 15: 730,047)	G → T (Glu → stop)						Illumina: 51% (38/74) Sanger: 50% (5/10)
	rtTA 562 (chr 15: 730,167)	T → C (Phe → Leu)						Illumina: 29% (28/95) Sanger: 40% (4/10)
24hr, r3	rtTA 13 (chr 15: 729,618)	G → T (Asp → Tyr)						Illumina: 21% (15/73) Sanger: 50% (5/10)
	rtTA 275 (chr 15: 729,880)	G → A (Ser → Asn)						Illumina: 8% (5/63) Sanger: 10% (1/10)
	rtTA 346 (chr 15: 729,951)	C → A (Gln → Lys)						Illumina: 1% (1/76)* Sanger: 10% (1/10)
	rtTA 651 (chr 15: 730,256)	78 bp deletion						Illumina: 0% Sanger: 30% (3/10)

Appendix Table S3. Mutations detected by whole-genome Illumina sequencing and targeted Sanger sequencing in conditions DiZ0 and D2Z0.

(A) Mutations in condition DiZ0. All columns are as indicated for **Appendix Table S2**.

(B) Mutations in condition D2Z0. All columns are as indicated for **Appendix Table S2**.

Experiment D0Z2	Location	Mutation	Read counts: Day 9	Read counts: Day 19
12hr, r1	*INO2* -159<br>*YDR124W* -688<br>(chr 4: 699,627)	A → deletion	Illumina: 2% (4/176)*<br>Sanger: NA	Illumina: 28% (21/75)<br>Sanger: 20% (2/10)
	YHR127W 613<br>(chr 8: 361,525)	G → A<br>(Ala → Thr)	Illumina: 7% (12/178)<br>Sanger: NA	Illumina: 8% (7/91)<br>Sanger: 0%
	GAL2 692<br>(chr 12: 290,903)	G → T<br>(Gly → Val)	Illumina: 2% (3/190)*<br>Sanger: NA	Illumina: 29% (26/89)<br>Sanger: 20% (2/10)
	GAL2 1006<br>(chr 12: 291,217)	T → A<br>(Phe → Ile)	Illumina: 5% (9/191)<br>Sanger: NA	Illumina: 14% (11/78)<br>Sanger: 20% (2/10)

Experiment D0Z2	Location	Mutation	Day 3	Day 9	Day 14	Day 19
24hr, r1	*YDR186C* 1742<br>(chr 4: 833,751)	T → A<br>(Synonymous)	Illumina: <1% (1/277)*<br>Sanger: NA	Illumina: 6% (5/89)<br>Sanger: 0%	Illumina: 8% (8/101)<br>Sanger: NA	Illumina: <1% (2/313)*<br>Sanger: 0%
	CHO2 2567<br>(chr 7: 805,006)	A → C<br>(His → Pro)	Illumina: 0%<br>Sanger: NA	Illumina: 1% (1/74)*<br>Sanger: NA	Illumina: 5% (6/119)<br>Sanger: NA	Illumina: 14% (45/316)<br>Sanger: NA
	Intergenic (just after RPS0A)<br>(chr 7: 921,790)	T → deletion	Illumina: 0%<br>Sanger: NA	Illumina: 0%<br>Sanger: NA	Illumina: 8% (11/134)<br>Sanger: NA	Illumina: 12% (37/302)<br>Sanger: 30% (3/10)
	SRX1 -93<br>*CYT2* -325<br>(NHP6A binding site)<br>(chr 11: 278,188)	T → A	Illumina: 0%<br>Sanger: NA	Illumina: 7% (7/100)<br>Sanger: 0%	Illumina: 12% (11/94)<br>Sanger: NA	Illumina: 1% (4/323)*<br>Sanger: 0%
	SIN4 2838<br>*YNL235C* 213<br>(chr 14: 209,767)	A → deletion<br>(frameshift)	Illumina: 3% (7/275)*<br>Sanger: NA	Illumina: 5% (5/91)<br>Sanger: NA	Illumina: 7% (9/125)<br>Sanger: NA	Illumina: <1% (1/267)*<br>Sanger: NA
	EOS1 603<br>(chr 14: 477,430)	A → C<br>(Synonymous)	Illumina: 0%<br>Sanger: NA	Illumina: 1% (1/92)*<br>Sanger: NA	Illumina: 8% (9/117)<br>Sanger: NA	Illumina: 11% (35/305)<br>Sanger: 40% (4/10)
	yEFGP::zeoR -219<br>(chr 15: 723,701)	42 bp deletion	Illumina: 0%<br>Sanger: NA	Illumina: 0%<br>Sanger: 80% (8/10)	Illumina: 0%<br>Sanger: NA	Illumina: 0%<br>Sanger: 60% (6/10)
	GUP2 1250<br>(chr 16: 190,403)	A → deletion<br>(frameshift, leads to premature stop)	Illumina: 4% (10/255)*<br>Sanger: NA	Illumina: 11% (12/106)<br>Sanger: 10% (1/10)	Illumina: 5% (5/96)<br>Sanger: NA	Illumina: <1% (1/309)*<br>Sanger: NA
24hr, r2	*yEFGP::zeoR* 831<br>(chr 15: 724,750)	G → A<br>(Synonymous)				Illumina: 29% (56/194)<br>Sanger: 70% (7/10)
24hr, r3	Illumina: None<br>Sanger: NA					

Appendix Table S4. Mutations detected by whole-genome Illumina sequencing and targeted Sanger sequencing in conditions D0Z2 (antibiotic only).

All columns are the same as for ***Appendix Table S2***.

(A) Experiment DiZ2	Location	Mutation	Read counts	
			Day 9	Day 19
12hr, r1	*STB3* -66 (chr 4: 793,955)	C → G	Illumina: 15% (15/97) Sanger: NA	Illumina: 17% (12/69) Sanger: 10% (1/10)
	GAL2 568 (chr 12: 290,779)	A → C (Ile → Leu)	Illumina: 27% (32/118) Sanger: NA	Illumina: 78% (63/81) Sanger: 80% (8/10)
	GAL2 1061 (chr 12: 291,272)	C → A (Thr → Asn)	Illumina: 29% (36/125) Sanger: NA	Illumina: 26% (23/88) Sanger: 20% (2/10)
	rtTA -261 (chr 15: 729,345)	42 bp deletion	Illumina: 0% Sanger: NA	Illumina: 0% Sanger: 70% (7/10)
12hr, r2	Illumina: NA Sanger: None			
12hr, r3	Illumina: NA Sanger: None			

(B) Experiment D2Z2	Location	Mutation	Read counts	
			Day 9	Day 19
12hr, r1	rtTA -15 (chr 15: 729,591)	C → G	Illumina: 13% (36/276) Sanger: NA	Illumina: 54% (63/116) Sanger: NA
	rtTA 225 (chr 15: 729,830)	G → C (Trp → Cys)	Illumina: 10% (25/252) Sanger: NA	Illumina: 48% (68/141) Sanger: NA

			Day 5	Day 9	Day 10	Day 12	Day 15	Day 18	Day 19
24hr, r1	rtTA -9 (chr 15: 729,597)	G → C	Illumina: 0% Sanger: NA	Illumina: 9% (11/127) Sanger: 40% (4/10)	Illumina: 11% (15/132) Sanger: NA	Illumina: 18% (24/132) Sanger: NA	Illumina: 65% (93/142) Sanger: NA	Illumina: NA Sanger: 70% (7/10)	Illumina: 51% (53/103) Sanger: NA
	rtTA 225 (chr 15: 729,830)	G → C (Trp → Cys)	Illumina: 1% (2/201)* Sanger: NA	Illumina: 79% (88/112) Sanger: 50% (5/10)	Illumina: 84% (123/146) Sanger: NA	Illumina: 79% (94/119) Sanger: NA	Illumina: 23% (39/166) Sanger: NA	Illumina: NA Sanger: 30% (3/10)	Illumina: 44% (40/91) Sanger: NA
	rtTA 329 (chr 15: 729,934)	A → C (Tyr → Ser)	Illumina: 3% (6/215)* Sanger: NA	Illumina: 5% (6/112) Sanger: 10% (1/10)	Illumina: 3% (4/143)* Sanger: NA	Illumina: 3% (4/152) Sanger: NA	Illumina: 0% Sanger: NA	Illumina: NA Sanger: 0%	Illumina: 0% Sanger: NA
	rtTA 431 (chr 15: 730,036)	G → T (Cys → Phe)	Illumina: 2% (3/182)* Sanger: NA	Illumina: 6% (6/101) Sanger: 0%	Illumina: 1% (2/159)* Sanger: NA	Illumina: 1% (2/145)* Sanger: NA	Illumina: 1% (2/162)* Sanger: NA	Illumina: NA Sanger: 0%	Illumina: 0% Sanger: NA
24hr, r2	rtTA 225 (chr 15: 729,830)	G → C (Trp → Cys)							Illumina: 53% (60/113) Sanger: NA
	rtTA 225 (chr 15: 729,830)	G → T (Trp → Cys)							Illumina: 42% (47/113) Sanger: NA
24hr, r3	rtTA 728 (chr 15: 730,333)	T → A (Leu → stop)							Illumina: 96% (68/71) Sanger: NA

Appendix Table S5. Mutations detected by whole-genome Illumina sequencing and targeted Sanger sequencing in conditions DiZ2 and D2Z2 (both antibiotic and inducer).

(A) Mutations in condition DiZ2. All columns are as indicated for ***Appendix Table S2****.*

(B) Mutations in condition D2Z2. All columns are as indicated for ***Appendix Table S2****.*

Appendix Figure S1

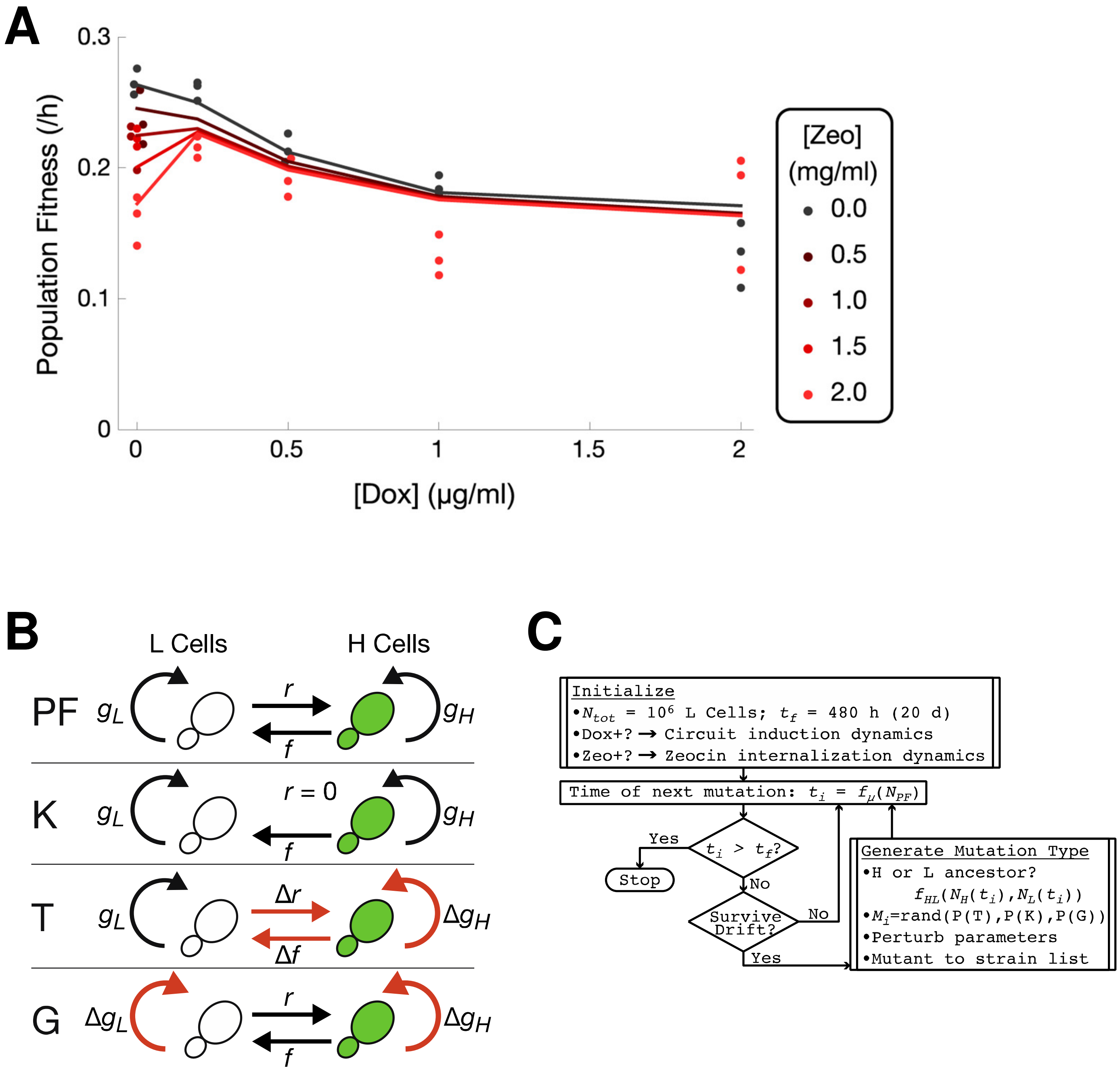

App. Fig. S2

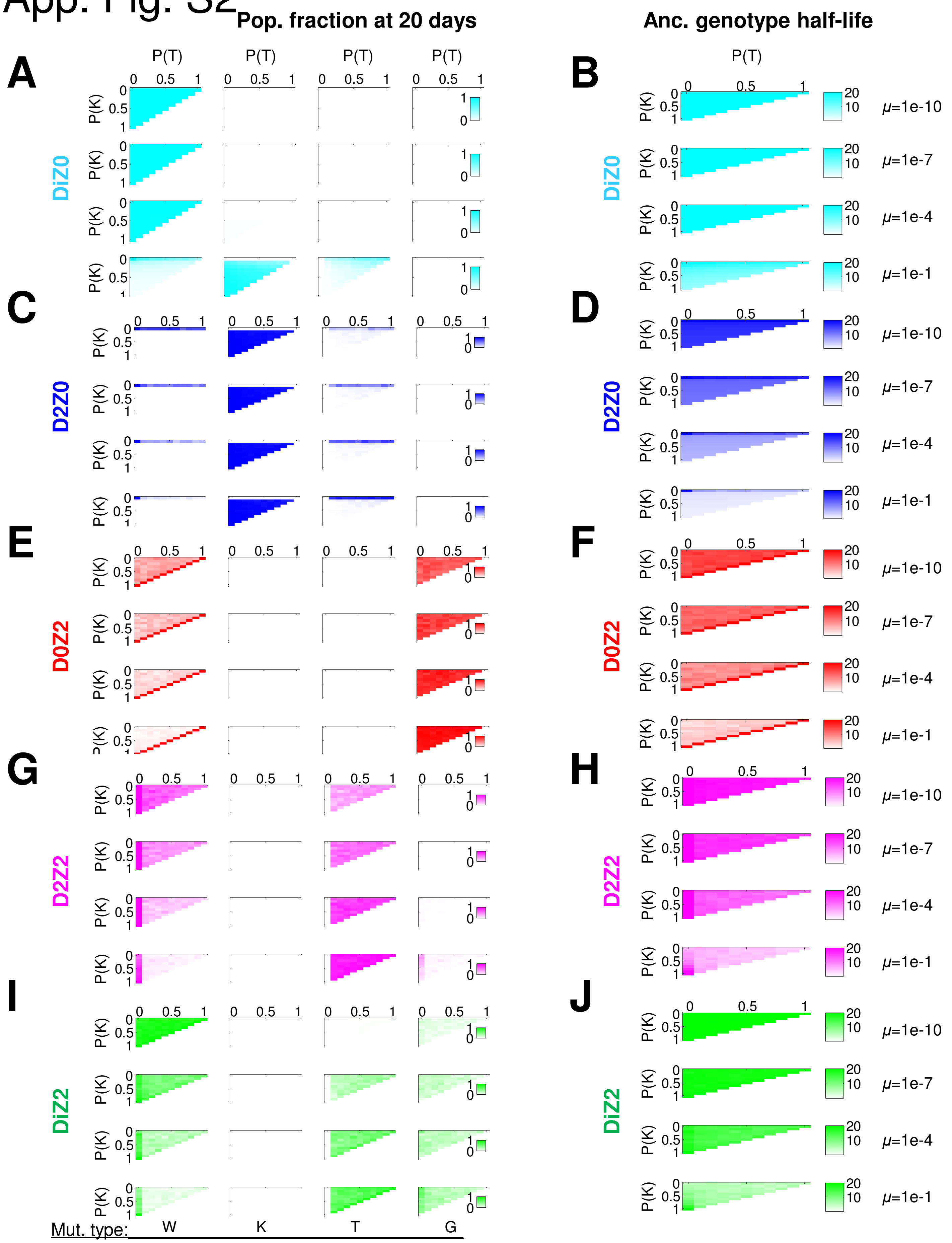

Pop. fraction at 20 days
Anc. genotype half-life
A
B
C
D
E
F
G
H
I
J
DiZ0
D2Z0
D0Z2
D2Z2
DiZ2
P(T)
P(K)
0
0.5
1
20
10
μ=1e-10
μ=1e-7
μ=1e-4
μ=1e-1
Mut. type:
W
K
T
G

Appendix Figure S3

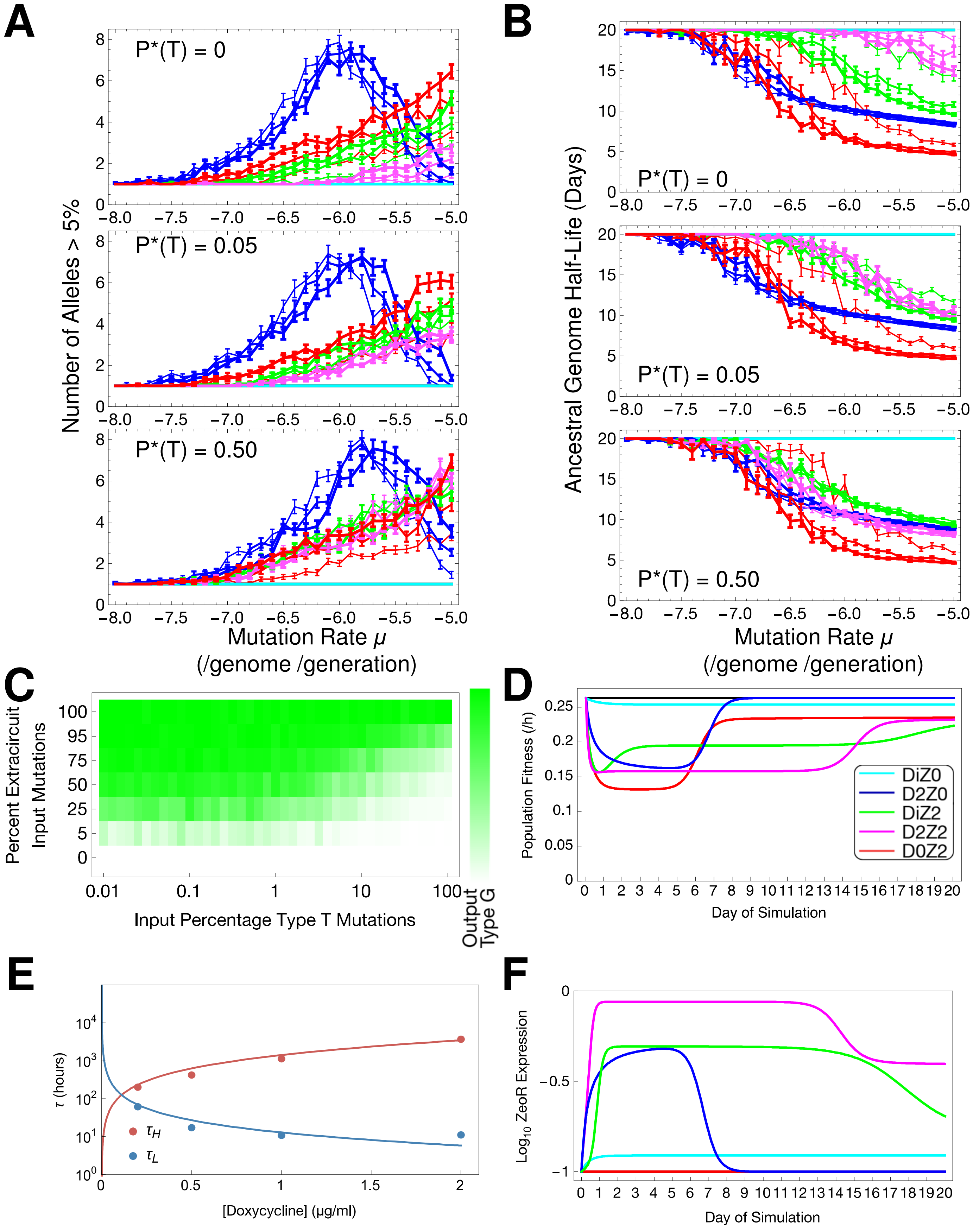

Appendix Figure S4

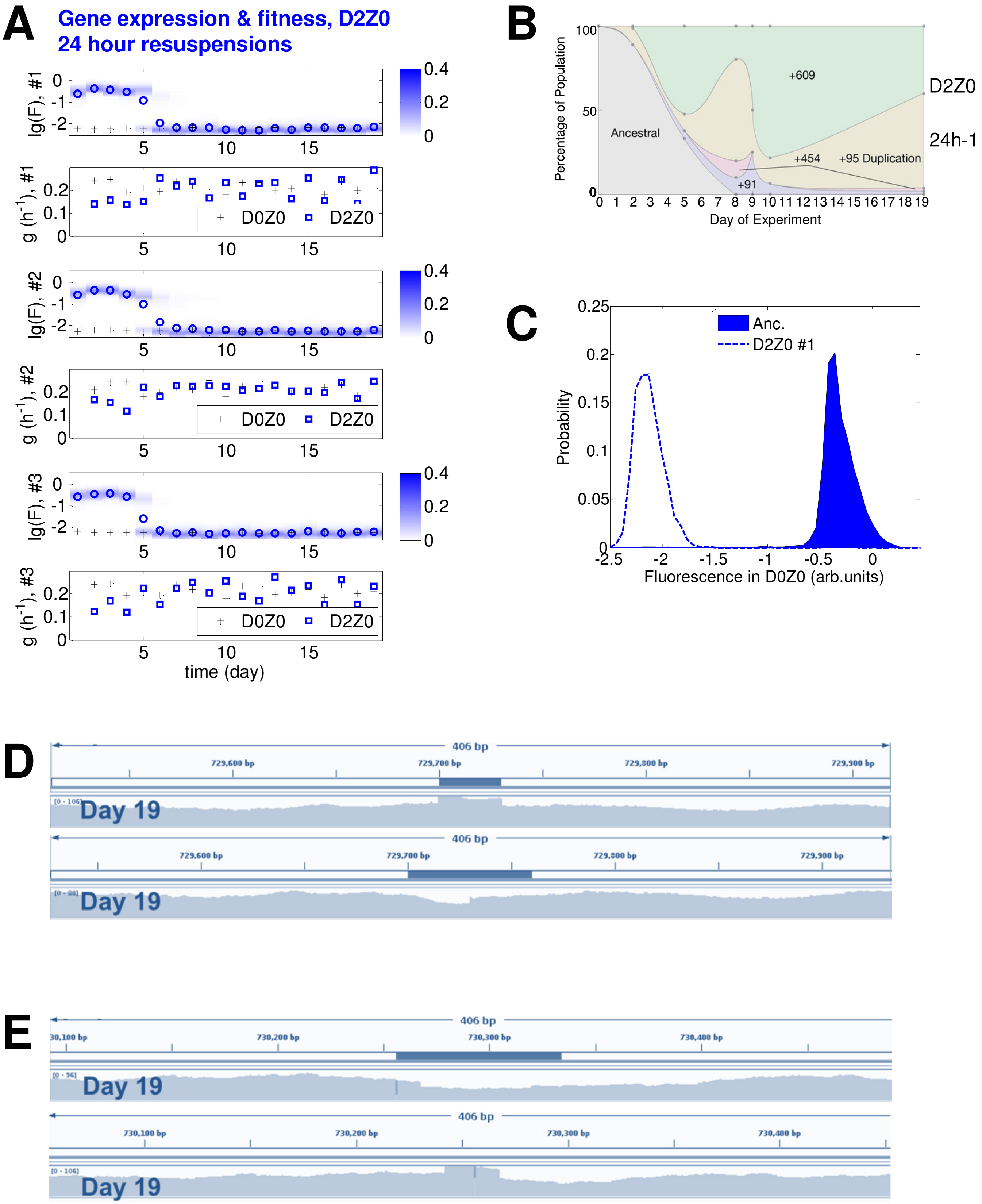

Appendix Figure S5

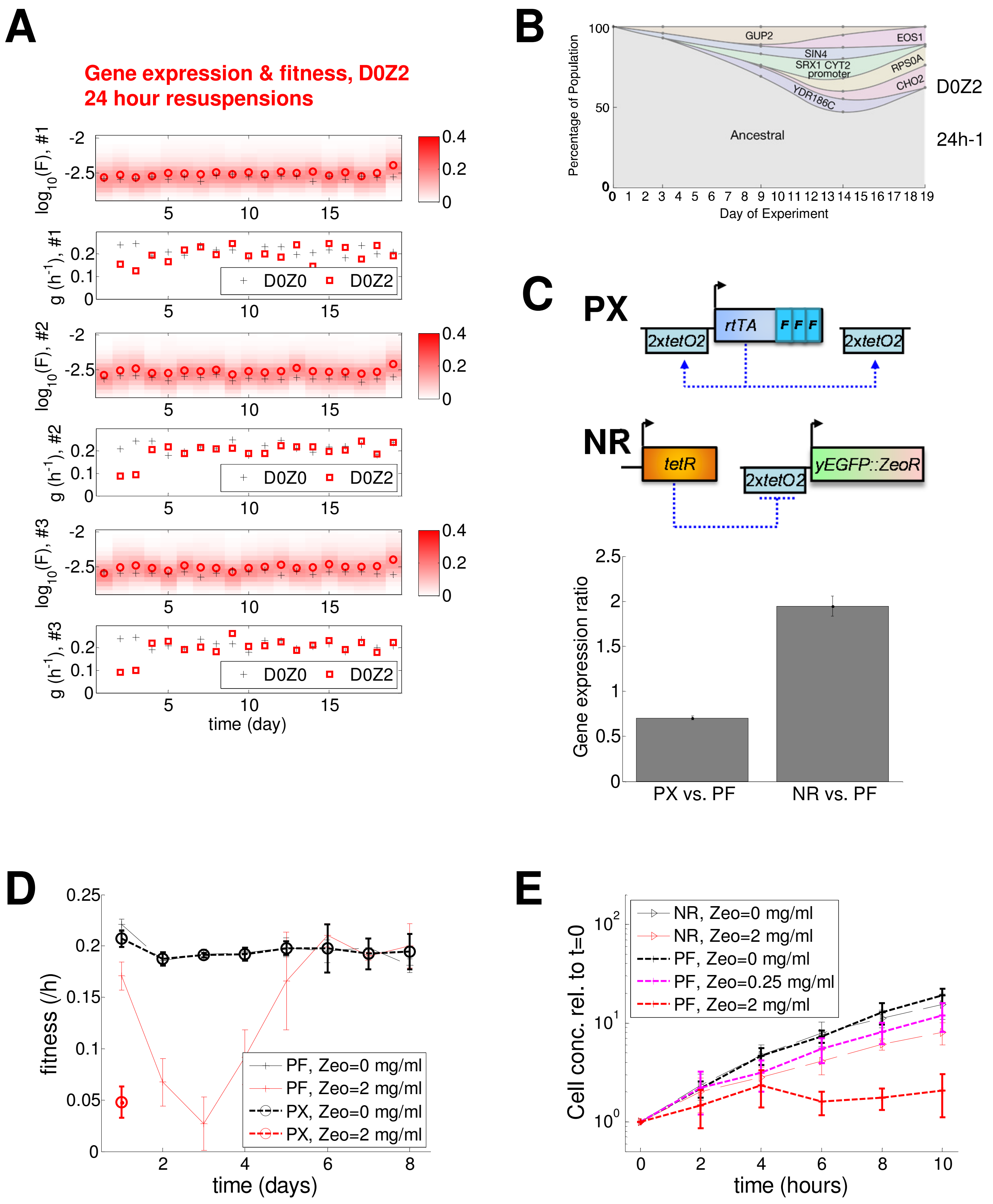

Appendix Figure S6

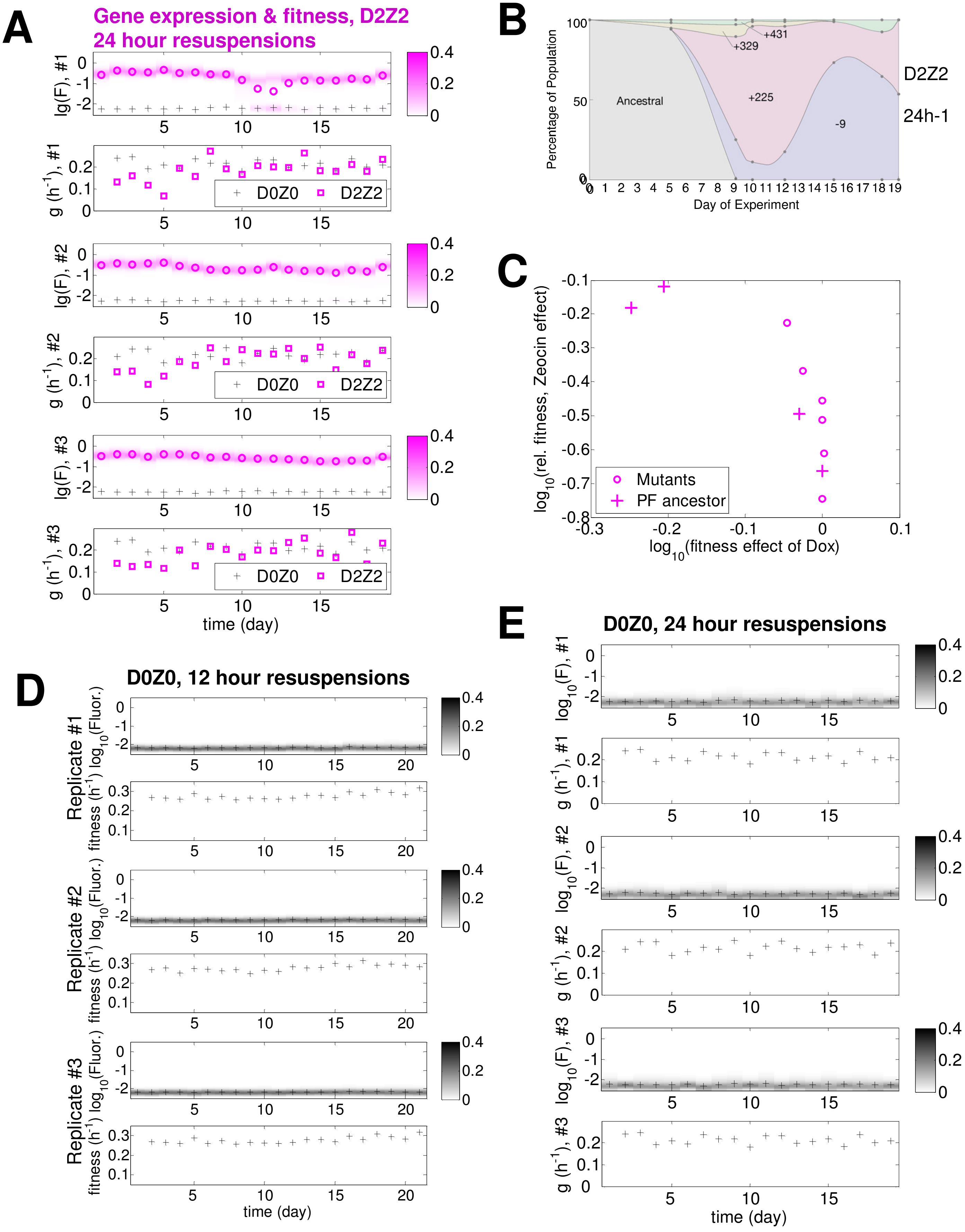

Appendix Figure S7

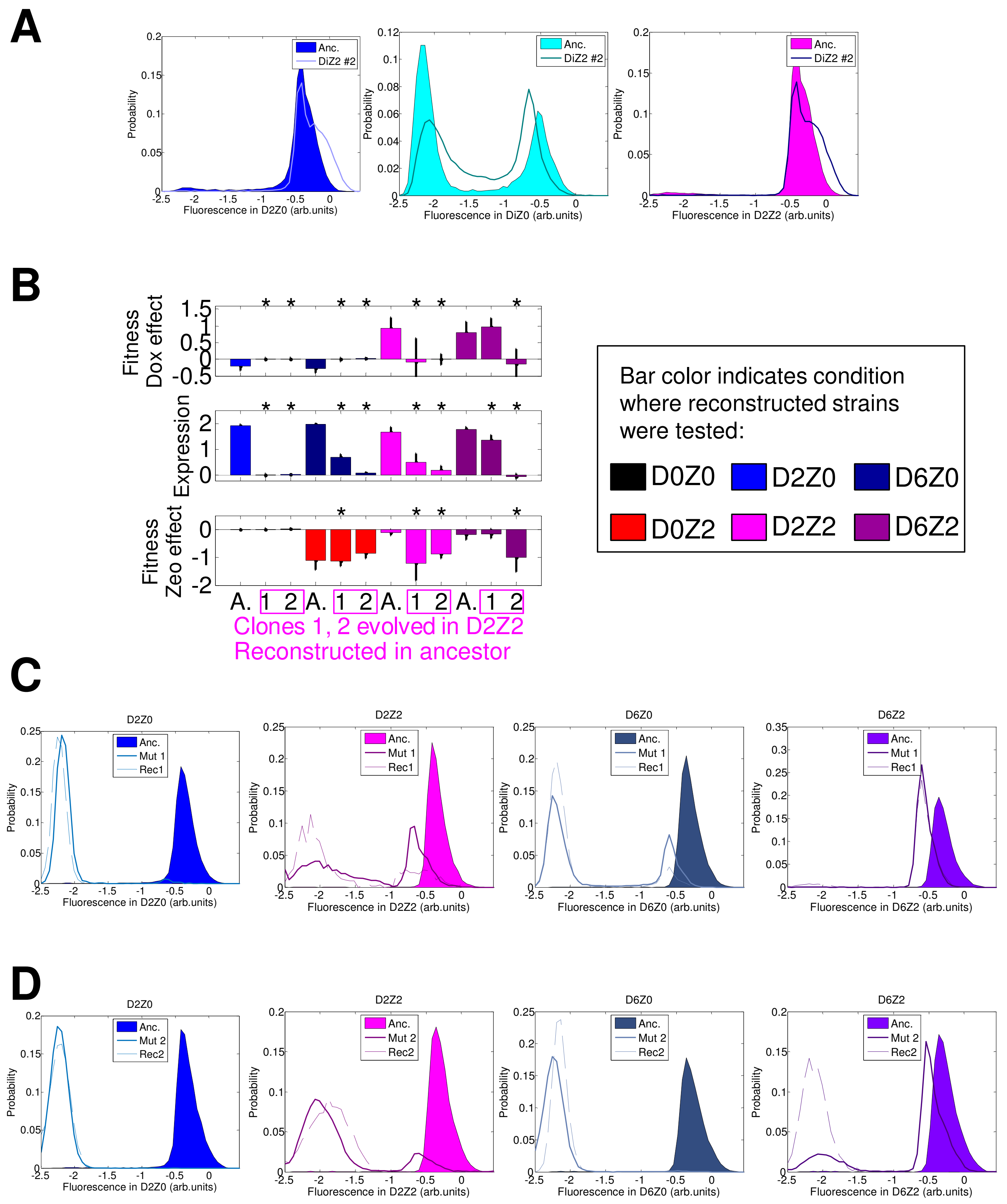

Appendix Figure S8

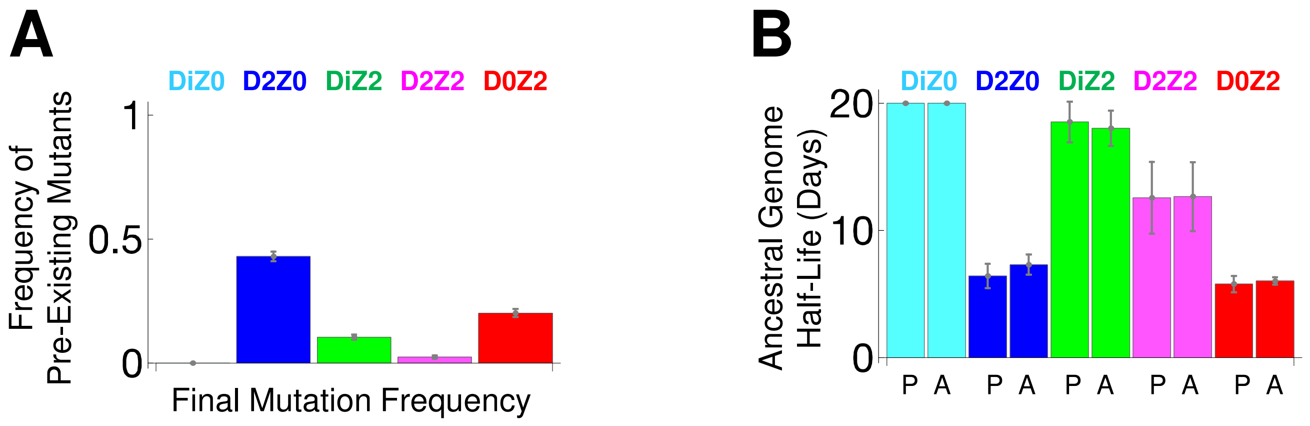

Supplement: Supplementary file 1 — Appendix [file msb0011-0827-sd1.pdf]
